# Genome-wide analysis of the FleQ direct regulon in Pseudomonas fluorescens F113 and Pseudomonas putida KT2440

**DOI:** 10.1038/s41598-018-31371-z

**Published:** 2018-09-03

**Authors:** Esther Blanco-Romero, Miguel Redondo-Nieto, Francisco Martínez-Granero, Daniel Garrido-Sanz, Maria Isabel Ramos-González, Marta Martín, Rafael Rivilla

**Affiliations:** 1 Departamento de Biología, Facultad de Ciencias, Universidad Autónoma de Madrid, Darwin, 2, 28049 Madrid, Spain; 2 Departamento de Protección Ambiental. Grupo de Microbiología Ambiental y Biodegradación, Estación Experimental del Zaidín, CSIC, Profesor Albareda, 1, 18008 Granada, Spain

## Abstract

Bacterial motility plays a crucial role in competitiveness and colonization in the rhizosphere. In this work, Chromatin ImmunoPrecipitation Sequencing (ChIP-seq) analysis has been used to identify genes putatively regulated by the transcriptional regulatory protein FleQ in *Pseudomonas fluorescens* F113 and *Pseudomonas putida* KT2440. This protein was previously identified as a master regulator of flagella and biofilm formation in both strains. This work has demonstrated that FleQ from both bacteria are conserved and functionally equivalent for motility regulation. Furthermore, the ChIP-seq analysis has shown that FleQ is a global regulator with the identification of 121 and 103 FleQ putative binding sites in *P. fluorescens* F113 and *P. putida* KT2440 respectively. Putative genes regulated by FleQ included, as expected, flagellar and motility-related genes and others involved in adhesion and exopolysaccharide production. Surprisingly, the ChIP-seq analysis also identified iron homeostasis-related genes for which positive regulation was shown by RT-qPCR. The results also showed that FleQ from *P. fluorescens* F113 shares an important part of its direct regulon with AmrZ, a global regulator also implicated in environmental adaption. Although AmrZ also regulates motility and iron uptake, the overlap occurred mostly with the iron-related genes, since both regulators control a different set of motility-related genes.

## Introduction

Flagella biosynthesis in pseudomonads requires more than 50 genes subjected to four levels of hierarchical regulation^1^. In this regulatory cascade, the transcriptional regulator FleQ appears to be the master regulator^2^. The function of FleQ in flagella synthesis regulation has been studied in *Pseudomonas aeruginosa*^1,2^, in *P. putida*^3–5^ and in *P. fluorescens*^6–8^. In these species, mutations in the *fleQ* gene result in non-motile, aflagellated bacteria. FleQ is an atypical enhancer binding protein (EBP) from the NtrC family of bacterial transcription factors (TFs) with three fundamental domains: a N-terminal REC domain which lacks the aspartic acid that serves as a phosphorylation site in other members of the same family, a central AAA+/ATPase σ^54^ (RpoN)-interaction domain and a C-terminal helix-turn-helix DNA-binding domain^9^. It has been described that FleQ is able to activate the expression of genes involved in flagellar export (*flhA* and *fliLMNOPQ* operon), localization and regulation of the flagellar apparatus (*flhF* and *fleN*), structural components of the flagellar basal body and motor switch complex (*fliEFG*) and the *fleSR* genes^2,10^. In the regulation of flagellar operons, FleQ works along with the alternative σ factor RpoN^2^. It also works together with the anti-activator FleN, another ATPase, by means of direct protein-protein interactions^11–15^. It has also been shown that FleQ can specifically bind the bacterial second messenger cyclic di-guanosine monophosphate (c-di-GMP)^16,17^ and the crystal structure of FleQ bound to c-di-GMP has been resolved^18^. Interestingly, most of the flagellar genes are moderately regulated by c-di-GMP, showing a downregulation when the intracellular level of this molecule is high^13^.

Besides the flagellar operons, FleQ regulates the biosynthesis of *P. aeruginosa* exopolysaccharides (EPSs, *pel* and *psl* operons) in a c-di-GMP-dependent manner, triggering either the activation or repression of these genes^14,18^. In the regulation of these operons, FleQ does not rely on RpoN but on the vegetative sigma factor (σ^70^)^14^. In the case of the *pel* operon the mechanism proposed incorporating structural and functional data implies that FleQ binds to two sites in the promoter of the operon but the effect on gene expression depends on the level of c-di-GMP. Without c-di-GMP, a hexamer of FleQ, although bound to two sites, relies on one of the sites (FleQ box 2) to repress gene expression when bound to FleN in presence of ATP. On the other hand, in response to c-di-GMP, the FleN/FleQ/DNA complex suffers a conformational change turning the FleQ multimer into an activator from the other promoter site (FleQ box 1)^14^. In addition, FleQ has been recently described as regulator of two strain-specific EPSs in *P. putida* KT2440, Pea and Peb^17^, the first being a key element of biofilm formation in this bacterium^19–21^. Other polysaccharide regulated by FleQ in a c-di-GMP dependent mode in *P. putida* is cellulose, through transcriptional regulation of the *bcs* operon^17,22^. FleQ has also been shown to regulate the expression of the *cdrA* and *lapA* genes, encoding adhesins required for biofilm formation in *P. aeruginosa*^23^ and *P. putida*^24^ respectively, in a c-di-GMP-dependent way. Furthermore, FleQ has been shown to be essential for biofilm formation in *P. putida*^25,26^.

Another central node in environmental adaption in pseudomonads is the transcriptional regulator AmrZ^27,28^. A ChIP-seq assay in *P. fluorescens* F113 showed that at least 215 genes were putatively regulated by AmrZ. AmrZ was shown to regulate genes required for iron homeostasis, synthesis and degradation of c-di-GMP and motility^28^. Similar results were obtained in *P. aeruginosa*^29^. AmrZ is an important determinant of c-di-GMP levels. In F113, AmrZ transcriptionally regulates multiple genes encoding diguanylate cyclases and the *amrZ* mutant shows enhanced motility, altered exopolysaccharides production, reduced biofilm formation, lack of rhizosphere colonization competence and reduced cytoplasmic levels of c-di-GMP^30^. It is important to note that AmrZ strongly represses the expression of the *fleQ* gene in both, *P. aeruginosa* and *P. fluorescens* species^27,31^.

Considering that FleQ and AmrZ regulate similar traits such as motility, exopolysaccharides production and biofilm formation, the aim of this work was to identify the genes and operons regulated by FleQ in *P. fluorescens* F113 and *P. putida* KT2440 by using ChIP-seq and to analyze the possible overlap between the FleQ and AmrZ regulons in *P. fluorescens* F113.

## Results

### FleQ from *P. fluorescens* F113 and *P. putida* KT2440 are functionally equivalent for motility regulation

**Figure 1:**
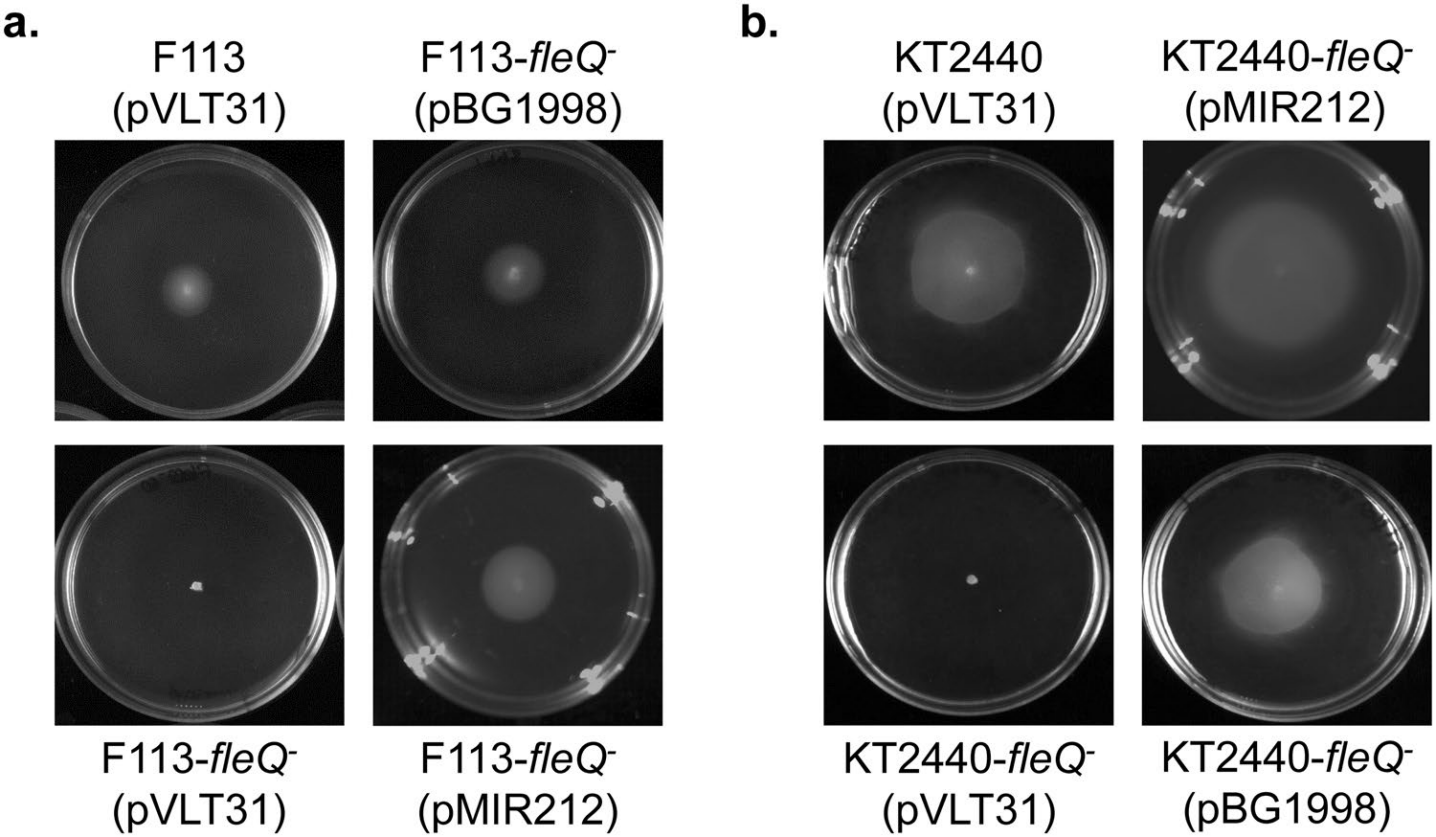
HA-FleQ proteins from *P. fluorescens* F113 and *P. putida* KT2440 are functionally equivalent in the regulation of flagella synthesis. Swimming motility of *Pseudomonas fluorescens* F113 WT and *fleQ* mutant harbouring the empty vector pVLT31, pBG1998 (pVLT31 HA-FleQ_F113_ construct) or pMIR212 (pVLT31 HA-FleQ_KT2440_ construct) (**a**). Swimming motility of *P. putida* KT2440 WT and its *fleQ* mutant harbouring the empty vector pVLT31, pBG1998 or pMIR212 (**b**). Swimming haloes produced in SA or LB with 0.3% (w/v) purified agar were observed 24–48 h after inoculation. Similar results were obtained with both media. Each experiment was done at least in triplicate. Typical results are shown.

FleQ is the master regulator for flagella synthesis in pseudomonads and *fleQ* mutants are non-motile because they lack flagella. In order to determine the functionality of HA-FleQ fusion proteins to be used for ChIP-seq, we complemented the swimming motility phenotype of *fleQ* mutant derivatives of F113 and KT2400 with their respective cloned fusion genes. As shown in Fig. 1, both HA-FleQ fusions were functional and were able to complement the motility defect of the *fleQ* mutants in both strains. Figure 1 shows that HA-FleQ_KT2440_ was also able to complement the motility of the F113 *fleQ* mutant and HA-FleQ_F113_ complemented the KT2440 *fleQ* mutant. These results show not only the functionality of the HA fusions, but also that FleQ proteins from both species are functionally equivalent, at least in the regulation of motility.

### FleQ is a global bifunctional transcriptional regulator in *P. fluorescens* F113 and *P. putida* KT2440

**Figure 2:**
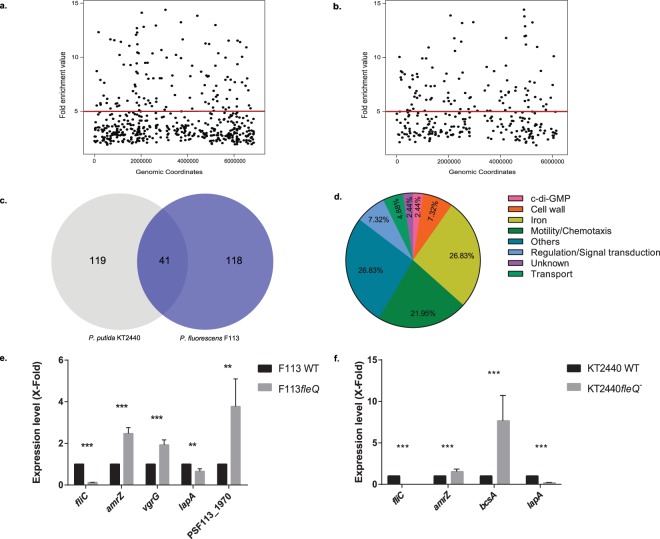
FleQ is a global transcriptional regulator that can act both as an activator and as repressor in *P. fluorescens* F113 and *P. putida* KT2440. FleQ binding sites distribution along the *P. fluorescens* F113 (**a**) and *P. putida* KT2440 genomes (**b**). Fold enrichment value for each of the peaks after peak calling with MACS 1.4 is represented against the coordinates in which these accumulations are located. Red line marks the five-fold enrichment threshold fixed in the analysis. Venn diagram representation of the genes predicted to be in FleQ_F113_ and FleQ_KT2440_ regulons (**c**). Pie chart depicting the functional classification of FleQ-regulated genes shared by *P. fluorescens* F113 and *P. putida* KT2440. Percentage of the 41 genes shared by both species in each functional category according to Gene Ontology database is represented (**d**). Genes included in these graphs are listed in Table 1. Gene expression analysis of putative FleQ-regulated genes by RT-qPCR assays in *P. fluorescens* F113 (**e**) and *P. putida* KT2440 (f). Expression level in the wild-type strain was considered 1 for each of the tested genes. Fold variation for each gene was determined by the 2^−ΔΔCT^ method. RNA was extracted after growth in SA medium to an O.D.600 ≈ 0.8. The asterisks denote statistically significant differences (**P < 0.01, ***P < 0.001) found with t-test for independent samples and Bonferroni-Dune method.

Four independent ChIP assays with N-tagged FleQ (HA-FleQ_F113_ in *Pseudomonas fluorescens* F113 and HA-FleQ_KT2440_ in *P. putida* KT2440) were performed, yielding 10 ng of immunoprecipitated DNA. Immunoprecipitated DNA from the four replicas was pooled and subjected to Illumina sequencing. After quality filtering, 6,653,248 reads (40.5% overall alignment rate) in the case of F113 and 20,558,997 (70.8% overall alignment rate) in the case of KT2440 of 50 nts length were used for subsequent experiments. In the case of F113, bioinformatic analysis yielded 496 peaks distributed along all the genome (Fig. 2a). Using a threshold of five-fold enrichment, 121 peaks were selected. Eighty-nine of these peaks (73.55%) were located in intergenic regions and 94.2% of them upstream an open reading frame (ORF). Gene assignment to peaks was done according to the nearest start codon. When two start codons were affected, both genes were selected as putative FleQ targets. In this way, 159 genes appeared as putatively affected by FleQ in F113. Similar results were obtained for KT2440. As shown in Fig. 2b, 279 peaks were also distributed along the chromosome. By using the same five-fold threshold, 103 peaks were selected. A percentage equal to 69.31% of them was also located in intergenic regions, 98% upstream an ORF, resulting in 160 genes likely regulated by FleQ in this strain. The genome-wide distribution of peaks and the overrepresentation of intergenic regions clearly indicate the role of FleQ proteins as global regulators. Supplementary Tables 1 and 2, list all the genes putatively affected by FleQ direct regulation in F113 and KT2440, respectively. As shown in Fig. 2c, an overlap of 41 promoter regions occur between the two species, indicating that these orthologues are potentially FleQ regulated in both species. Furthermore, 56.1% of these common genes corresponded to genes implicated in motility, iron homeostasis and cell wall formation (Fig. 2d), indicating the similar roles of the FleQ proteins in *P. fluorescens* and *P. putida*. Genes identified in this study common to both species are listed in Table 1.

**Table 1:** List of genes predicted to be regulated by FleQ both in *P. fluorescens* F113 and in *P. putida* KT2440.

FUNCTIONAL CLASS	LOCUS	GENE	PRODUCT
c-di-GMP	PSF113_5738/ PP_5263	*—*	GGDEF/EAL domains containing protein
CELL WALL	PSF113_0208/ PP_0168	*lapA*	Surface adhesion protein
	PSF113_4136/ PP_1970	*—*	Lipoprotein
	PSF113_4752 PP_1288	*algD*	GDP-mannose 6-dehydrogenase
IRON	PSF113_1274/ PP_1006	*—*	TonB-dependent hemoglobin/transferrin/lactoferrin family receptor
	PSF113_2454/ PP_3086	*—*	ECF family RNA polymerase sigma-70 factor
	PSF113_2456/ PP_2590	*—*	Outer membrane ferric siderophore receptor
	PSF113_2589/ PP_4606	*—*	T ferric siderophore receptor
	PSF113_3151/ PP_4755	*—*	TonB-dependent siderophore receptor
	PSF113_3153/ PP_0704	*—*	ECF subfamily RNA polymerase sigma factor
	PSF113_4568/ PP_1083	*—*	BFD(2Fe-2S)-binding domain-containing protein
	PSF113_4845/ PP_4611	*—*	ECF family RNA polymerase sigma-70 factor
	PSF113_4896/ PP_3325	*—*	Outer membrane ferric siderophore receptor
	PSF113_5412/ PP_0350	*—*	Ferrichrome-iron receptor
	PSF113_5691/ PP_0180	*—*	Cytochrome C family protein
MOTILITY/CHEMOTAXIS	PSF113_0569/ PP_4888	*—*	Methyl-accepting chemotaxis sensory transducer
	PSF113_1531/ PP_4386	*flgF*	Flagellar basal body rod protein FlgF
	PSF113_1532/ PP_4385	*flgG*	Flagellar basal body rod protein FlgG
	PSF113_1562/ PP_4370	*fliE*	Flagellar hook-basal body protein FliE
	PSF113_1582/ PP_4344	*flhA*	Flagellar biosynthesis protein FlhA
	PSF113_1583/ PP_4343	*flhF*	Flagellar biosynthesis regulator FlhF
	PSF113_4454/ PP_4391	*flgB*	Flagellar basal-body rod protein FlgB
	PSF113_4456/ PP_4393	*cheV-3*	Chemotaxis protein CheV
	PSF113_4457/ PP_4394	*flgA*	Flagellar basal body P-ring biosynthesis protein FlgA
OTHERS	PSF113_0351/ PP_5059	*—*	Hypothetical protein
	PSF113_0572/ PP_4880	*vacB*	Ribonuclease R
	PSF113_0711/ PP_4674	*recC*	Exodeoxyribonuclease V subunit gamma
	PSF113_1201/ PP_1638	*fpr*	Oxidoreductase FAD/NAD(P)-binding domain-containing protein
	PSF113_1592/ PP_4334	*—*	ParA family protein
	PSF113_1815/ PP_2239	*rhtA*	Cysteine transporter
	PSF113_4204/ PP_1878	*—*	Hypothetical protein
	PSF113_4567/ PP_1084	*—*	Anti-oxidant AhpCTSA family protein
	PSF113_5315/ PP_0437	*birA*	Biotin-protein ligase
	PSF113_5482/ PP_4960	*fda*	Fructose-1,6-bisphospate aldolase
	PSF113_5739/ PP_5264	*rep*	ATP-dependent DNA helicase Rep
REGULATION/SIGNAL TRANSDUCTION	PSF113_1200/ PP_1637	*—*	LysR family transcriptional regulator
	PSF113_1897/ PP_1978	*—*	TetR family transcriptional regulator
	PSF113_4470/ PP_4470	*amrZ*	Arc domain-contaning protein DNA binding domain-containing protein
TRANSPORT	PSF113_0210/ PP_0167	*—*	LapA Type I secretion system ATPase
	PSF113_1510/ PP_4519	*tolC*	TolC type I secretion outer membrane protein
VIRULENCE	PSF113_5053/ PP_0685	*—*	Hypothetical protein

Gene expression analysis was performed in both species for a selected group of genes that have a peak in their promoter region. As shown in Fig. 2e,f all the tested genes showed regulation by FleQ. As expected, FleQ acts as a bifunctional regulator, activating the expression of genes implicated in motility and adhesion (*fliC, lapA*) and as a repressor for the expression of genes implicated in exopolysaccharides production (*bcsA*, PSF113_1970) and others, both in *P. fluorescens* F113 and in *P. putida* KT2440. Interestingly, *amrZ* was negatively regulated by FleQ both in *P. fluorescens* F113 and in *P. putida* KT2440.

Since regulation by FleQ may be influenced by the second messenger c-di-GMP, similar ChIP-seq experiments to those reported above were performed in F113 and KT2440 backgrounds with altered c-di-GMP levels. For F113, a *bifA*^*-*^ background was used for high c-di-GMP levels and a *sadC*^*-*^*wspR*^*-*^ for low levels of the second messenger^32^. A *bifA*^*-*^ background was used in KT2440 for elevated c-di-GMP in comparison to the WT^33^. The results were not significantly different from those in the wild-type strains. In the case of F113, 126 out of the 159 genes identified as putatively regulated in the wild-type strain, were also identified in the *bifA*^*-*^ assay and 106 in the *sadC*^*-*^*wspR*^*-*^ assay. One hundred and eighty are common between *bifA*^*-*^ and *sadC*^*-*^*wspR*^*-*^, that have extreme levels of c-di-GMP. In the case of *P. putida* KT2440, in the *bifA*^*-*^ background, 149 peaks with a fold enrichment higher than 5 were detected. 107 genes were coincident with the genes in the wild-type assay. These genes include all of the genes implicated in c-di-GMP turnover and exopolysaccharide production and most of the genes implicated in motility and iron homeostasis. It also included genes such as *lapA* and *amrZ*. Furthermore, ten other genes that appeared as potential FleQ targets in the wild-type assay, were also identified in the *bifA*^*-*^ background, although the peaks ranged between four and five-fold. These results clearly show that c-di-GMP does not play a relevant role in the binding of FleQ to promoters which is in agreement to the current proposed model of regulation^18^. Supplementary Tables 3–5, list the genes identified as putatively regulated by FleQ in each of these genetic backgrounds.

### Regulation of motility by FleQ is conserved in *P. fluorescens* F113 and *P. putida* KT2440

**Figure 3:**
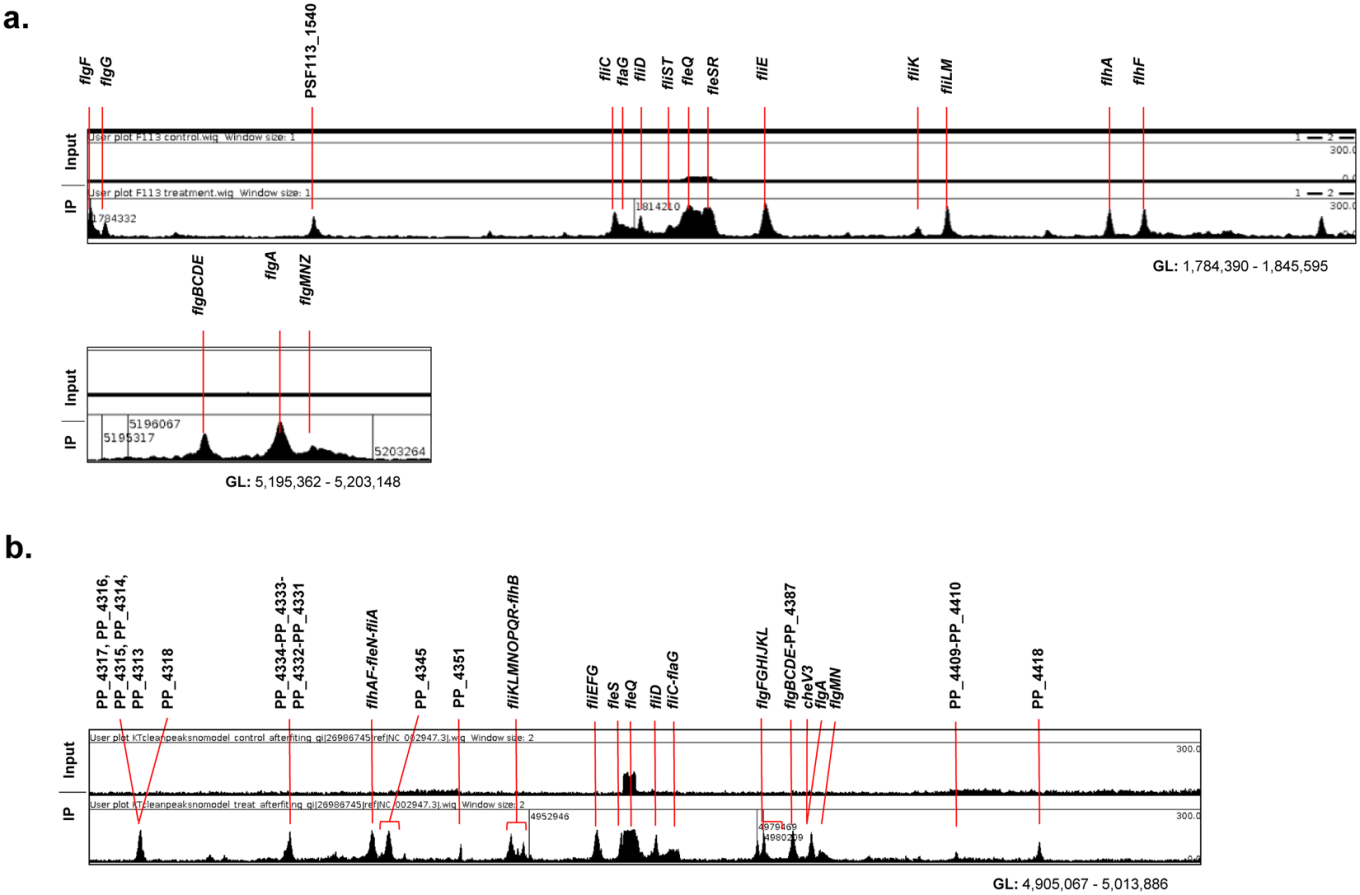
FleQ binds to promoters of genes included in the flagellar gene clusters from *P*. F113 and *P. putida* KT2440. HA-FleQ immunoprecipitation (IP) reads were plotted against the number of reads from the non-immunoprecipitated DNA (Input). Regions represented correspond to the flagellar gene clusters of both strains: nts 1,784,390 to 1,845,595 and nts 5,195,362 to 5,203,148 in the case of *P. fluorescens* F113 (**a**) and from nt 4,905,067 to 5,013,886 in *P. putida* KT2440 (**b**). The genes or gene clusters presenting a peak in its promoter are marked on the top of the graph. Artemis Sanger release 16.0.0 genome viewer was used for the representation.

As expected, among the selected genes we found the flagellar regions, known to be regulated by FleQ, in both species. Our results show that regions regulated by FleQ such as the flagellar regulon, contain more binding sites than those detected in the bioinformatics analysis, due to a masking effect produced by overlapping regions (Fig. 3) or the stringent cut-off used. Considering the two main flagellar regions in the F113 genome, 11 genes were assigned according to MACS output (Supplementary Table 1). A closer look at these regions (Fig. 3a) showed that 17 genes/gene clusters are likely regulated by FleQ: *flgF, flgG*, PSF113_1540, *fliC*, *flaG*, *fliD*, *fliST*, *fleQ*, *fleSR*, *fliE*, *fliK, fliLM, flhA, flhF* (Fig. 3a; nts 1,784,390 to 1,845,595) and *flgBCDE*, *flgA* and *flgMNZ* region (Fig. 3a; nts 5,195,362 to 5,203,148). Despite the different organization of the flagellar region in KT2440 (Fig. 3b), mostly the same genes/gene clusters than in F113 are likely regulated by FleQ, indicating a very similar regulatory pattern in both species. The only remarkable difference is the lack of a peak upstream the *fliST* genes in KT2440. These results are consistent with the interchangeable functions of both *fleQ* alleles shown in Fig. 1.

### FleQ activates the expression of iron homeostasis genes in *P. fluorescens* F113 and *P. putida* KT2440

**Figure 4:**
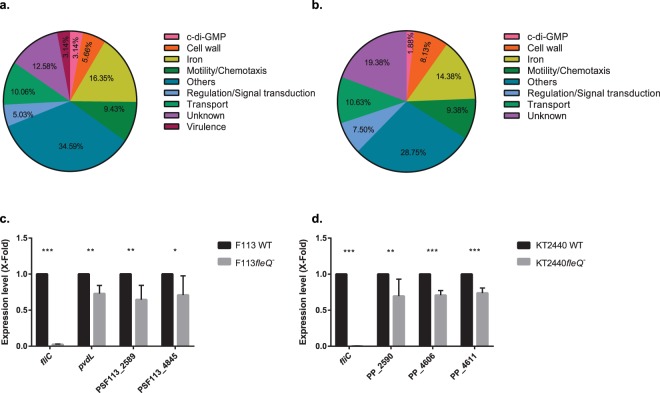
FleQ is involved in the activation of iron homeostasis-related genes in *P. fluorescens* F113 and *P. putida* KT2440. Pie chart representation of the genes likely controlled by FleQ in *P. fluorescens* F113 (**a**) and *P. putida* KT2440 (**b**) divided in functional classes. Genes found in ChIP-seq analysis according to Gene Ontology database and percentages are shown. Genes included in this graph are listed in Supplementary Table 1 (**a)** and Supplementary Table 2 (**b**). Gene expression analysis of iron homeostasis-related genes in *P. fluorescens* F113 (**c**) and *P. putida* KT2440 (**d**) by RT-qPCR. Selected iron homeostasis-related genes predicted to be regulated by FleQ were tested in both *P. fluorescens* F113 (*pvdL*, PSF113_2589 and PSF113_4845) (**c**) and *P. putida* KT2440 (PP_2590, PP_4606 and PP_4611) (**d**). Relative expression of the genes in *fleQ* mutants compared to WT strains grown in CAS medium supplemented with bipyridyl for F113 and SA for KT2440 is represented. RNA was extracted at O.D.600 of 0.8. The asterisks denote statistically significant differences (*P<0.05, **P < 0.01, ***P < 0.001) with t-test analysis for independent samples and Bonferroni-Dune method.

After assignation of the selected peaks in both species (Supplementary Tables 1 and 2), genes were classified in different functional categories according to the Gene Ontology database. As shown in Fig. 4a,b there was an overrepresentation of genes related to “iron homeostasis” (16.35%, 14.38%), “motility/chemotaxis” (9.43%, 9.38%), “regulation and signal transduction” (5.03%, 7.50%) and “cell wall” (5.66%, 8.13%) in both species, representing together one third of the genes putatively regulated by FleQ.

Other represented classes include “transport” (10.06%, 10.63%), “c-di-GMP” (3.14%, 1.88%) and “virulence” (3.14%, 0%). The remaining genes were included in “others” category (34.59%, 28.75%) or their functions are unknown (12.58%, 19.38%). In order to test the relevance of FleQ as a regulator of iron homeostasis, we analyzed the expression of several iron related genes in iron deficient conditions, in both backgrounds, in the wild-type strain and in the *fleQ* mutant. As shown in Fig. 4c,d, FleQ influences the transcription of these genes as an activator in both species.

### DNA consensus binding sequence for FleQ remains undetermined

With the purpose of finding a specific motif binding site for FleQ, the summit positions of the 121 peaks in the F113 ChIP-seq assay and 103 peaks for KT2440 and a region of 100 nts on each side were introduced in the MEME tool. However, it was not possible to determine a robust motif for FleQ as many peaks were located in the promoter region of iron-responding genes in both cases. Therefore, the main resulting motif was the iron responsive Fur-box motif (not shown). This motif was present in 44 of the peaks with an e-value of 1.5e^−034^ in F113. To avoid iron bias those regions containing a Fur motif were removed from the dataset, resulting in 62 peaks that were, again, analyzed with the same tools. Once more for this particular situation, it was not possible to obtain a FleQ DNA-binding consensus motif, as sequences corresponding to the IHF and σ^54^ binding sites masked any other possible conserved sequence. Similar results were found with the KT2440 peaks. In view of the fact that no conserved region for FleQ binding site could be found, a FleQ consensus sequence (GTCaNTAAAtTGAC) that has been proposed for *P. aeruginosa*^23^, was searched with MAST, BLASTn and FIMO in *P. fluorescens* F113. The genes that included this *P. aeruginosa* motif, such as *lapA*-like, PSF113_1970, *fliL*, *fliE* and *flhA*, were found in our F113 analysis and corresponded to peaks. However, a total of 536 matches were detected in the F113 genome, 490 of them with a *p*-value below the 1e-5 range (the same value published for this motif in the selected sequences from *P. aeruginosa*). Most of the matches corresponded to regions that were not present in our ChIP-seq output. Consequently, we were not able to define a robust FleQ consensus binding sequence in *P. fluorescens* F113. More recently, a consensus sequence for the FleQ binding site in *P. putida* KT2440 (GTCAaAAAAtTGAC) was proposed^17^ based on the promoter regions of 15 selected genes and the previously proposed consensus for *P. aeruginosa*. The genes included *fliE*, *lapA*, *algD*, *bcs*, *pea*, *peb*, *fleS*, *flhA*. Similarly to F113, the FleQ binding site in KT2440 was searched in the pool of 103 peaks obtained in the KT2440 ChIP-seq assay using FIMO and MAST tools. As a result, 18 matches were found (*p*-value < 0.0001) and were attributed to 15 peaks, as in some peaks the motif appeared more than once. The 15 peaks were assigned to 15 genes. Five genes (29.5%) were classified in the “cell wall” category while the remaining 10 genes were distributed in “motility/chemotaxis” (*fliE* and *flhB*), “iron”, “transport”, “regulation/signal transduction” (*amrZ*), “others” and “unknown” categories. Genes included in “cell wall” functional class were *lapA, algD* and genes of the *pea*, *peb and bcs operons*, all of them previously identified *in vitro*^17^. The same coincidence was observed with *fliE*. The motif was found three times in the case of *lapA* and twice in *pea*. Although this motif seems to be congruent in a specific set of genes, being most of them related with exopolysaccharide synthesis, we were unable to propose a consensus sequence that might expand to a majority of the genes identified as being regulated by FleQ in *P. putida* KT2440. Evaluation of intergenic peaks independently did not provide further information in the search for the union consensus sequence in any of the strains.

### FleQ and AmrZ share an important part of their direct regulon in *P. fluorescens* F113

**Figure 5:**
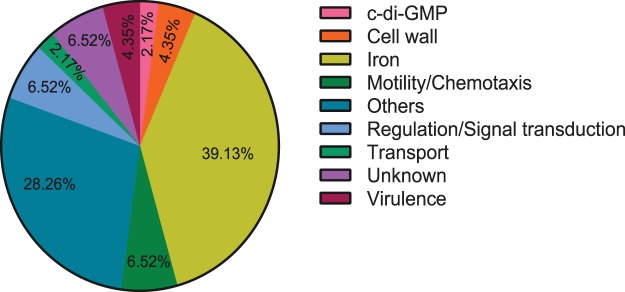
FleQ and AmrZ share part of their regulons in *P. fluorescens* F113. Pie chart showing the genes predicted to be regulated both by FleQ and AmrZ in the strain F113. Classification in functional categories of the 45 shared genes according to Gene Ontology database is represented. Genes included in this graph are listed in Table 2.

As shown above, the *amrZ* gene appears to be repressed by FleQ both in *P. fluorescens* and in *P. putida*. Since AmrZ has been shown to be a global motility and iron regulator in F113, the 159 genes putatively regulated by FleQ in this strain were compared with the 215 genes found to be putatively regulated by AmrZ^28^. The results showed an overlap of 45 genes putatively regulated by both proteins in *P. fluorescens* F113 (Table 2). Overlap occurred in genes related to “iron” (39.13%), followed by “motility/chemotaxis” (6.52%), “regulation/signal transduction” (6.52%), unknown functions (6.52%), “virulence” (4.35%), “cell wall” (4.35%), “c-di-GMP” (2.17%) and “transport” (2.17%) (Fig. 5). It is important to notice that most of the iron uptake genes found in the AmrZ regulon are also present in the FleQ regulon. This is not the case for the motility/chemotaxis-related genes, where the overlap is small and the two transcriptional regulators seem to regulate a different set of genes (Table 2).

**Table 2:** List of genes predicted to be regulated by both FleQ and AmrZ in *P. fluorescens* F113.

FUNCTIONAL CLASS	LOCUS	GENE	PRODUCT
c-di-GMP	PSF113_4023	*—*	Diguanylate cyclase phosphodiesterase with PAS/PAC sensor
CELL WALL	PSF113_0208	*lapA*	LapA
	PSF113_4752	*algD*	GDP-mannose 6-dehydrogenase
IRON	PSF113_0933	*fagA*	FagA
	PSF113_1274	*—*	TonB-dependent hemin, ferrichrome receptor
	PSF113_1322	*—*	Iron-regulated protein A precursor
	PSF113_1749	*pvdS*	PvdS
	PSF113_1750	*pvdL*	PvdL
	PSF113_1837	*pvdD*	PvdD
	PSF113_1856	*—*	Outer membrane pyoverdine eflux protein
	PSF113_2258	*—*	Outer membrane ferripyoverdine receptor
	PSF113_2454	*—*	RNA polymerase sigma-70 factor, ECF subfamily
	PSF113_2589	*—*	Ferrichrome-iron receptor
	PSF113_3151	*—*	Ferrichrome-iron receptor
	PSF113_3220	*—*	Heme uptake regulator
	PSF113_3734	*—*	Ferrichrome-iron receptor
	PSF113_4045	*—*	Iron-regulated membrane protein
	PSF113_4568	*—*	Bacterioferritin-associated ferredoxin
	PSF113_4845	*—*	RNA polymerase sigma-70 factor, ECF subfamily
	PSF113_5412	*fiuA*	FiuA
	PSF113_5657	*fbpA*	FbpA
MOTILITY/CHEMOTAXIS	PSF113_0569	*—*	Methyl-accepting chemotaxis protein
	PSF113_0751	*flhD*	FlhD
	PSF113_2159	*—*	Methyl-accepting chemotaxis protein
OTHERS	PSF113_0079c	*—*	Phage-related replication protein-like protein
	PSF113_1047	*—*	Multicopper oxidase
	PSF113_1201	*—*	Ferredoxin–NADP( + ) reductase
	PSF113_2126	*—*	Dihydrodipicolinate synthase
	PSF113_2158	*nuoA*	NuoA
	PSF113_2972	*—*	Glycosaminoglycan degradation
	PSF113_3889	*—*	Zinc carboxypeptidase domain protein
	PSF113_3918	*tig*	Tig
	PSF113_3922	*folD*	FolD
	PSF113_4083	*—*	Sterol desaturase
	PSF113_4204	*—*	Protein binding
	PSF113_4932	*prs*	Prs
	PSF113_4978	*—*	Pentapeptide repeat-containing protein
REGULATION/SIGNAL TRANSDUCTION	PSF113_1200	*—*	LysR family transcriptional regulator
	PSF113_4024	*—*	Transcriptional regulator, Cro/CI family
	PSF113_4470	*amrZ*	AmrZ
UNKNOWN	PSF113_2272	*—*	Hypothetical protein
	PSF113_2273	*—*	Reticulocyte binding protein
	PSF113_5053	*—*	Hypothetical protein
VIRULENCE	PSF113_1855	*—*	RHS repeat-associated core domain-containing protein
	PSF113_2409	*vgrG*	VgrG

## Discussion

In this work, we have characterized the direct regulon of the master regulatory protein FleQ in *P. fluorescens* F113 and *P. putida* KT2440. FleQ is an EBP present in all pseudomonads and related bacteria. In this study FleQ has been revealed as a global regulator implicated in the regulation of gene expression of probably more than one hundred genes/gene clusters in both species. FleQ binding sites were distributed along the genomes and the majority of them were located in intergenic regions, showing a strong bias of the binding of FleQ towards intergenic regions, where most of the promoters are placed. Distribution of binding sites is similar in both species and a significant number of orthologues are putatively regulated by FleQ both in F113 and in KT2440. Gene ontology classification of putative FleQ regulated genes is also very similar. Furthermore, the cross-complementation of the swimming motility phenotypes of *fleQ* mutants with heterologous alleles, shown in Fig. 1, strongly indicates that *fleQ* genes play similar roles in both species. Although c-di-GMP has been shown to play an important role in regulation by FleQ, we have shown here that binding of this regulator to promoters is independent of the levels of the second messenger in both species, since the FleQ binding sites identified are very similar in strains with inactivated DGC/PDE. In this sense, Xiao *et al*.^22^, showed that in *P. putida* KT2440 binding of FleQ to the *gcbA* promoter was inhibited by high levels of c-di-GMP. However, our results show binding of FleQ to the *gcbA* promoter both in the wild-type strain and in the *bifA* mutant, which is in agreement with the observation made for FleQ binding to *pel* promoter independently of the presence of c-di-GMP^18^. The discrepancy about reported for *gbcA* could well be due to the different type of experiment performed: an *in vitro* binding assay versus an *in vivo*, although not physiological experiment.

We have been unable to determine a consensus sequence for the binding of FleQ even when only interspecific peaks were included in the bioinformatic analysis. Over the years, different attempts and *in vitro* techniques have been carried out trying to define a DNA-binding consensus sequence for FleQ, but no consistent results have been obtained. DNase I footprinting has been performed in order to determine FleQ binding sites on the promoters of a set of flagellar genes^10^. Though no conserved binding site was found, two different acting ways were suggested for FleQ: activation from a distance with a looping in the *fleSR* promoter and binding in the vicinity of the promoter without looping in the case of *flhA, fliE* and *fliL*^10^. Furthermore, attempts to find reproducible DNase I footprints of FleQ at the flagellar promoter *fleSR* were unsuccessful^14^. More recent studies also using this approach were able to define a motif for the binding of this protein to a limited set of promoters in *P. aeruginosa*^23^. However, we have shown here that this motif, although present in a few of the detected peaks, is also present hundreds of times in other non-enriched regions of the genome and is therefore unreliable, at least in F113. Different is the case of the consensus sequence proposed in *P. putida* KT2440^17^ which resulted too stringent as to include most of the experimentally FleQ-enriched sequences.

FleQ was originally described as the master regulator of flagella synthesis in *P. aeruginosa*^2^ and *fleQ* mutants are non-motile^11,12^. It has been shown that it is required for the expression of flagellar genes, including *fleSR*, *flhA, fliE, and fliL*^10^. The role of FleQ in the regulation of flagella synthesis has also been shown in other pseudomonads, such as *P. fluorescens*^6–8^, *P. chlororaphis*^34^ and *P. syringae*^35^. The results presented here have confirmed the direct role of FleQ in the regulation of flagellar genes in *P. fluorescens* and *P. putida* and FleQ binding has been shown *in vivo* to promoters of the flagellar region, such as *fliC*, *fliE*, *fliL*, *flhA, flhD* and *flhF*, among others. The role of FleQ as an activator of flagella synthesis in these two bacteria was also evident by the lack of expression of the *fliC* gene in a *fleQ* mutant background in both species and by the complementation of swimming defects of *fleQ* mutants.

FleQ has also been found to be a negative regulator of the expression of genes with a role in exopolysaccharide synthesis in *P. aeruginosa*. It has been previously shown that FleQ repressed the expression of the *pel* genes involved in the synthesis of the Pel exopolysaccharide and that this repression was reversed by c-di-GMP^13^. Similarly, the expression of the *psl* genes, required for the Psl exopolysaccharide is also regulated in a c-di-GMP dependent way in the same bacterium^23^. *Pseudomonas fluorescens* F113 does not produce any of these polysaccharides. However, a peak was detected in the promoter region of PSF113_1970, the first gene of an operon likely to encode the genes for the synthesis of a specific EPS not produced by *P. aeruginosa* or *P. putida*. We have also shown that the expression of PSF113_1970 is higher in a *fleQ* mutant background, indicating that the synthesis of this putative EPS is negatively regulated by FleQ. It has been shown previously that strain KT2440 produces four exopolysaccharides: alginate (*alg*), cellulose-like (*bcs*) and two less characterized polysaccharides, Pea and Peb^21^. It is known that FleQ binds *in vitro* to the promoter region of the gene clusters encoding the synthesis of Bcs, Pea and Peb and that this regulator strongly represses the synthesis of alginate under cell wall stress conditions^17^. Our *in vivo* experiments have validated the binding of FleQ to the *bcs*, *alg* and *peb* promoters, since peaks have been detected in these locations. We have not detected a peak upstream of PP_3132, the first gene in the *pea* operon. However, a strong peak was found close to this location, upstream of PP_3126, encoding a “polysaccharide biosynthesis/export protein” that might have a double functionality in the production of the Pea polysaccharide. We have also confirmed in this work that FleQ is a strong repressor of *bcsA* expression. Regarding biofilm formation, FleQ has been shown to positively regulate the expression of the *lapA* gene, which encodes a large adhesin essential for biofilm formation. Positive regulation of *lapA* and *lapA*-like genes has been shown in *P. putida*^24^ and *P. aeruginosa*^23^. We have confirmed here the *in vivo* binding of FleQ to the *lapA* promoter and the transcriptional activation of this gene by the regulator in *P. putida*. We have extended this observation to *P. fluorescens* by showing that in F113, FleQ also binds to the *lapA* promoter and that *lapA* is transcriptionally activated by FleQ.

We have also found that FleQ is likely to regulate genes and operons in *P. fluorescens* F113 and *P. putida* KT2440 that have not been previously identified as regulated by FleQ in any pseudomonad. Among these genes some are related with c-di-GMP turnover, regulation, transport and notably iron homeostasis. Gene ontology analysis showed that genes with similar functions are putatively regulated by FleQ in both species, showing again the functional homology of FleQ proteins. Regarding iron, we have shown binding of FleQ to the *pvd* promoters, responsible for the synthesis of the major siderophore pyoverdine in F113. Peaks in the regions of these promoters have also been found in the KT2440 genome, although at a lower significance value than in F113 (not shown). Many other iron-responsive genes implicated in iron uptake also showed binding of FleQ in their promoter regions in both species. Expression analysis of several of these genes in F113, KT2440 and theirs *fleQ* mutant backgrounds, under iron limitation, has shown that deprived of iron these genes are under positive regulation by FleQ in both species. These results determine a novel role for FleQ proteins in pseudomonads, as positive regulators of iron homeostasis.

**Figure 6:**
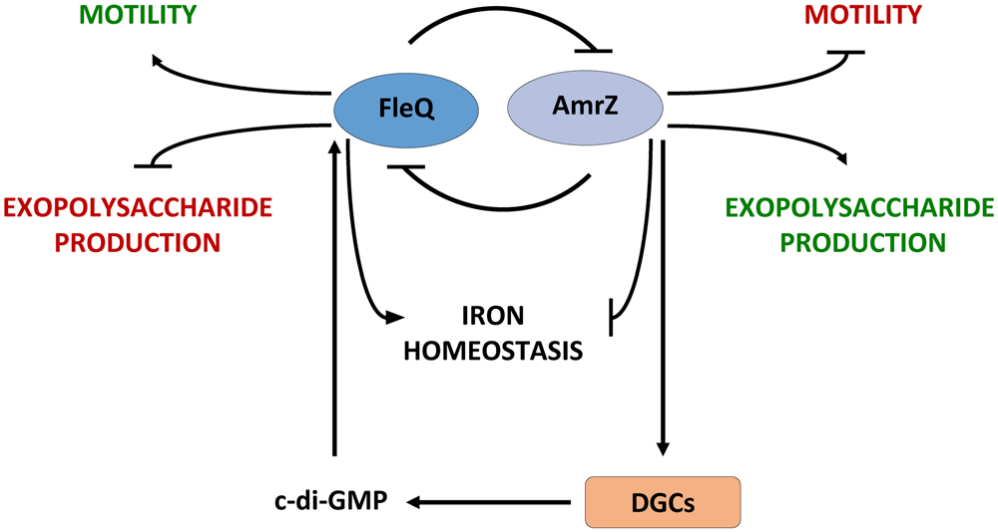
FleQ and AmrZ form a central hub for environmental adaption in *P. fluorescens* F113. Proposed model of the FleQ and AmrZ interplay in the regulation of traits implicated in environmental adaption. According to this model, FleQ and AmrZ form an oscillator by its mutual transcriptional repression. FleQ acts as an activator of motility and expression of iron homeostasis genes and as a repressor of exopolysaccharide genes. Conversely, AmrZ activates EPSs production genes and represses motility and iron homeostasis genes. The second messenger c-di-GMP participates in this circuit, since AmrZ activates the expression of diguanylate cyclases and FleQ transcriptional regulation is modulated by c-di-GMP binding.

Other novel gene regulated by FleQ in *P. fluorescens* F113 and *P. putida* KT2440 is *amrZ*. In our ChIP-seq analysis with FleQ, enriched regions have been found upstream of the *amrZ* gene in both species. Furthermore, gene expression analysis has shown that *amrZ* is under transcriptional repression by FleQ. It is important to notice that *fleQ* itself is under strong AmrZ repression both in *P. aeruginosa*^31^ and *P. fluorescens*^27^. Since AmrZ is also a major regulator of motility and biofilm formation and has been found to negatively regulate iron homeostasis genes in *P. aeruginosa*^29^ and *P. fluorescens* F113^28^, we decided to compare the AmrZ and the FleQ direct regulons in F113. We have found that 45 genes are putatively regulated by both TFs. Among these genes it is noteworthy that almost every iron-related gene that is directly regulated by AmrZ, is also directly regulated by FleQ. For these iron homeostasis genes, AmrZ acts as a weak repressor^28^ and FleQ as a weak activator, showing therefore opposing roles. If AmrZ and FleQ interact between them or whether they compete for same regions in the promoter of these genes is currently unknown. In the case of motility and exopolysaccharides genes, most are regulated either by AmrZ or by FleQ although a few of them are overlapping genes. The function of this reciprocal regulation is unclear, although it could work as an oscillator, as indicated in Fig. 6. In this model FleQ and AmrZ work as a central hub for environmental adaption. AmrZ would be a negative regulator of motility and iron homeostasis genes and a positive regulator of exopolysaccharide production. FleQ would play an opposing role, by activating iron homeostasis genes and motility, but repressing exopolysaccharides production. We have recently shown that AmrZ is a major regulator of c-di-GMP levels in F113, by activating several diguanylate cyclases^30^. FleQ in turn has been shown to bind c-di-GMP in *P. aeruginosa*^13,16^ and *P. putida*^17^ and it was revealed that this binding determines its transcriptional activity.

Genome wide analysis of the FleQ regulon have been previously performed by microarray hybridization in *P. fluorescens* strains Pf0-1^36^ and SBW25^37^. Although these strains and F113 belong to the *P. fluorescens* complex of species, they have been shown to be different species, belonging to different phylogenetic groups^38^. In both cases, Pf0-1 and SBW25, more than one hundred genes were shown to be differentially expressed in the wild-type strain compared to the *fleQ* mutant. These differentially expressed genes belong to different functional classes, showing that in these strains, FleQ is also a global regulator. Although many genes are common with genes reported here to be putatively regulated by FleQ in F113 and *P. putida* KT2440, iron homeostasis related genes were not found to be regulated by FleQ in Pf-01 or SBW25. However, in the case of SBW25, experiments were performed in iron-sufficient medium (LB) where iron uptake genes are under strong repression by Fur and are not expressed. Furthermore, the level of activation reported in our studies for several of these genes, would have been below the threshold used in both microarrays studies. This indicates that ChIP-seq is valuable in identifying entire regulons of master regulators.

FleQ is an atypical EBP. Being a global regulator, it acts both as a transcriptional activator and as a repressor. Its activity depends on the levels of the second messenger c-di-GMP. Conversely to other EBPs, its activation does not depend on phosphorylation by an histidine kinase^18^ and it seems to be able to function with different sigma factors: σ^54^ in the case of most flagellar genes^10^, σ^28^ in the case of *fliC*^8^, and σ^70^ in the case of biofilm genes^14^. In this sense, it has been proposed a role for FleQ in interplay with c-di-GMP and several sigma factors in determining *P. putida* life-style, switching between flagellar motility and biofilm formation^4^. On the other hand, FleQ is widely conserved among the pseudomonads and seems to regulate similar genes. The results presented here describe for the first time the implication of FleQ in the regulation of iron homeostasis. Whether FleQ might interact with the subfamily of extracytoplasmic function (ECF) sigma factors in the regulation of iron homeostasis is subject of current investigation. All in all, the results presented in this work allow to conclude that together with AmrZ, FleQ is an important determinant for environmental adaption.

## Methods

### Bacterial strains, growth conditions, antibiotics and plasmids

In this study, four *P. fluorescens* F113 strains were used, a WT strain (F113Rif)^39^ a *fleQ* mutant constructed in this work by homologous recombination using pK18*mobsacB* vector^40^, a *bifA*^*-*^ and a *sadC*^*−*^*wspR*^*−*^ strains^32^. Additionally, three *P. putida* KT2440 strains were used, a WT strain, which is a plasmid-free derivative of *P. putida* mt-2^41^, a *fleQ* mutant^26^ and a *bifA*^*−*^strain^33^. F113, KT2440 and derivatives were grown at 28 °C in Luria-Bertani (LB) medium^42^ for the ChIP-seq experiments, sucrose-asparagine (SA)^38^ or LB media for swimming with *P. fluorescens* F113 and *P. putida* KT2440 and RT-qPCR assays with *P. putida* KT2440 or CAS medium (3.18 mM Ca(NO_3_)_2_, 1 mM MgSO_4_, 50 mM PIPES, pH adjusted to 6.8, containing 1 mM K_2_HPO_4_, 1% (w/v), casamino acids, and 1% (w/v) glycerol) supplemented with 100 µM 2,2’-bipyridyl (low-Fe)^43^ for RT-qPCR analysis with *P. fluorescens* F113. *Escherichia* coli strain DH5α (Gibco-BRL) carrying the appropriate plasmids for each conjugation was grown in LB medium at 37 °C. When solid growth medium was used, 1.5% (w/v) of purified agar was added. Antibiotics were supplemented to maintain or select for plasmids and mutants as follows: ampicillin (Amp) at 100 µg/mL, kanamycin (Km) at 50 µg/mL for *P. fluorescens* F113 or 25 µg/mL for *P. putida* KT2440, tetracycline (Tet) at 10 µg/mL for *E. coli* and 10–25 µg/mL for *P. fluorescens* F113 and *P. putida* KT2440.

The hemagglutinin peptide YPYDVPDYA (HA) was fused in-frame to the FleQ protein N-terminal domain by PCR using the primer: HAFleQ (5′-ATGTCTTATCCATACGATGTTCCAGATTATGCTTGGCGTGAAACCAAAATTC-3′) and FleQR (5′-TCAATCATCCGCCTGTTCAT-3′) in the case of *P. fluorescens* F113 and HAFleQ (5′-ATGTCTTATCCATACGATGTTCCAGATTATGCTTGGCGTGAAACCAAGATT-3′) and FleQR (5′-AAGCTTAATCCTCCGCCTGGTC-3′) for *P. putida* KT2440. The amplified fragments were cloned into the IPTG-inducible expression vector pVLT31^44^ to generate the plasmids pBG1998 and pMIR212, respectively.

### Complementation of the *fleQ* mutants

The functionality of the HA-FleQ fusion constructs used in the ChIP-seq experiments was validated by the restoration of motility in the *fleQ* mutants of *P. fluorescens* F113 and *P. putida* KT2440. Mutant complementation was done by introducing the recombinant plasmid pVLT31 either empty or carrying HA-FleQ into the corresponding mutant strain by triparental mating as reported previously^26^.

### Swimming motility assays

Motility of either WT F113 or KT2440, *fleQ* mutants and complemented *fleQ* mutants was tested by swimming assays. Cells were inoculated in triplicate in SA or LB media on 50 mm diameter plates containing 0.3% (w/v) purified agar by introducing a toothpick with the strain to be analyzed from previous solid cultures for F113 and liquid cultures for KT2440 and incubated at 28 °C. Haloes were observed after 24 h (LB) or 48 h (SA) incubation.

### ChIP-seq assay

Protein-DNA interaction and binding sites of FleQ were surveyed by Next-Generation Sequencing technology combined with chromatin immunoprecipitation (ChIP). In the ChIP experiment, transcriptions factors were cross-linked to DNA in their native state and immunoprecipitated (IP) following the experimental procedure as detailed previously^28^. In this experiment, 20 mL of LB-cultures at OD_600_ of 0.5 from *P. fluorescens* F113 and derivatives (pBG1998) and *P. putida* KT2440 and derivatives (pMIR212) were induced for 3 h with 0.1 mM IPTG^28^. Before immunoprecipitation a sample was prepared to be used as input in order to detect non-specific binding against the IP sample. Samples from four independent cultures per case were inmunoprecipitated and the DNA pooled. Sequencing of DNA samples was carried out by UT Health Science Center at San Antonio Genome Sequencing Facility using Illumina HiSeq. 3000 System single end (50 bp each read).

### Bioinformatic analysis

In order to remove Illumina adapters and low quality reads, sequences were clipped and filtered with Trimmomatic^45^, defining a sliding window of 4 nucleotides (nts) with an average Phred quality of 20 and 50 nts as minimum read length to be conserved.

With the aim to equalize the number of reads between input and IP samples, several steps were performed. A draft alignment with *P. fluorescens* F113 or *P. putida* KT2440 reference genome from GenBank (NC_016830 and NC_002947.4 respectively) was carried out using Bowtie v2^46^. Then, unmapped reads were cleaned with SamTools^47^ and Picard tools 2.4.1^48^. Reads number from input and IP files were equalized by random subsampling (n = 3) using an own designed Python script. Subsequently, a final alignment was conducted with Bowtie v2. Peak calling was done with MACS 1.4.2^49^ comparing input and IP files and specifying a q-value or false discovery rate (FDR) of 0.01. Peak distribution was visualized in Artemis release 16.0.0^50^ with the purpose of assigning a gene to each peak. Peaks with a fold-enrichment equal or greater than five were selected for *P. fluorescens* F113 data (Supplementary Tables 1, 3 and 4) and *P. putida* KT2440 (Supplementary Tables 2 and 5). Gene Ontology database^51^ was used to classify the genes into functional categories.

Regarding the search of a conserved motif model, 100 nts to both sides of each peak summit position were extracted using an own designed Python script and analyzed using MEME Suite 4.11.2^52^. Thus, consensus sequences were searched using MEME with a maximum length of 17 nts and compared to known transcription factor binding sites with TomTom. Obtained and already described motifs models were examined in the whole *P. fluorescens* F113 and *P. putida* KT2440 genomes with MAST, FIMO or BLASTn algorithms^53^ and contrasted with the sequences of the peaks with FIMO.

### RNA isolation, cDNA synthesis and gene expression analysis

Total RNA from F113, KT2440 and the *fleQ* mutant strains, grown in SA liquid medium to an O.D._600_ of 0.8, was extracted from 1 mL culture samples. Additionally, total RNA from F113 and its *fleQ* mutant grown in CAS medium supplemented with 100 µM of the iron-chelator 2,2′-bipyridyl to an O.D._600_ of 0.9 was obtained. Samples were subsequently centrifuged (14,000 × *g*, 2 min) at RT and supernatants were discarded. Then, 100 µL of RNAlater (Ambion, Waltham, MA, USA) was added to the cell pellets and these conserved at 4 °C.

RNA isolation was performed following the instructions of SV Total RNA Isolation System (Promega). Concentration and quality of the samples was determined using Nanodrop^®^ spectrophotometer. RNA integrity was confirmed in 0.8% (w/v) denaturing agarose gels. In addition, genomic DNA contamination in the samples was discarded by PCR (95 °C for 3 min, followed by 30 cycles of 95 °C for 30 s, 60 °C for 1 min, 72 °C for 1 min, and a final extension at 72 °C for 7 min) with the primers designed for RT-qPCR experiments (Supplementary Table 6).

Complementary DNA (cDNA) synthesis by reverse transcription (RT-PCR) was performed using Superscript IV^®^ Reverse Transcriptase (Invitrogen). Then, qPCR reactions of the cDNA synthesized were carried out in quadruplicate for each gene, using FastStart Universal SYBR Green Master Rox (Roche).

For both strains, two biological replicates were considered and gene expression was calculated using threshold cycle (Ct) values. Data was normalized by using *16S rRNA* expression as housekeeping and relativized to F113 or KT2440 WT following the 2^−ΔΔCt^ method^54^.

### Statistical analysis

R Commander^55^ and VennDiagram^56^ package in R software^57^ was used in the representation of the plot for genomic distribution of peaks and Venn diagram respectively. GraphPad Prism version 7.00 for Windows (GraphPad Software, La Jolla California USA, www.graphpad.com) was used in the statistical analysis and representation of RT-qPCR data, the comparison was done using multiple t-test for independent samples (p < 0.05) with Bonferroni-Dune method; and for the representation of the pie charts.

## Electronic supplementary material

Supplementary Information

## Data Availability

ChIP-seq raw data have been deposited to the NCBI Sequence Read Archive database and it is available under the accession number SRP145465.

## References

[1] Dasgupta N (2003). A four-tiered transcriptional regulatory circuit controls flagellar biogenesis in *Pseudomonas aeruginosa*. Molecular microbiology.

[2] Arora SK, Ritchings BW, Almira EC, Lory S, Ramphal R (1997). A transcriptional activator, FleQ, regulates mucin adhesion and flagellar gene expression in *Pseudomonas aeruginosa* in a cascade manner. Journal of bacteriology.

[3] Kieboom J, Bruinenberg R, Keizer-Gunnink I, De Bont JA (2001). Transposon mutations in the flagella biosynthetic pathway of the solvent-tolerant *Pseudomonas putida* S12 result in a decreased expression of solvent efflux genes. FEMS Microbiol Lett.

[4] Jimenez-Fernandez A (2016). Complex interplay between FleQ, cyclic diguanylate and multiple sigma factors coordinately regulates flagellar motility and biofilm development in *Pseudomonas putida*. PLoS One.

[5] Wang Y, Li Y, Wang J, Wang X (2017). FleQ regulates both the type VI secretion system and flagella in *Pseudomonas putida*. Biotechnol Appl Biochem.

[6] Robleto EA, López-Hernández I, Silby MW, Levy SB (2003). Genetic analysis of the AdnA regulon in *Pseudomonas fluorescens*: nonessential role of flagella in adhesion to sand and biofilm formation. Journal of bacteriology.

[7] Capdevila S, Martínez-Granero FM, Sánchez-Contreras M, Rivilla R, Martín M (2004). Analysis of *Pseudomonas fluorescens* F113 genes implicated in flagellar filament synthesis and their role in competitive root colonization. Microbiology.

[8] Redondo-Nieto M (2008). Transcriptional organization of the region encoding the synthesis of the flagellar filament in *Pseudomonas fluorescens*. Journal of bacteriology.

[9] Bush M, Dixon R (2012). The role of bacterial enhancer binding proteins as specialized activators of sigma54-dependent transcription. Microbiology and molecular biology reviews: MMBR.

[10] Jyot J, Dasgupta N, Ramphal R (2002). FleQ, the major flagellar gene regulator in *Pseudomonas aeruginosa*, binds to enhancer sites located either upstream or atypically downstream of the RpoN binding site. Journal of bacteriology.

[11] Dasgupta N, Arora SK, Ramphal R (2000). *fleN*, a gene that regulates flagellar number in *Pseudomonas aeruginosa*. Journal of bacteriology.

[12] Dasgupta N, Ramphal R (2001). Interaction of the antiactivator FleN with the transcriptional activator FleQ regulates flagellar number in *Pseudomonas aeruginosa*. Journal of bacteriology.

[13] Hickman JW, Harwood CS (2008). Identification of FleQ from *Pseudomonas aeruginosa* as a c-di-GMP-responsive transcription factor. Molecular microbiology.

[14] Baraquet C, Murakami K, Parsek MR, Harwood CS (2012). The FleQ protein from *Pseudomonas aeruginosa* functions as both a repressor and an activator to control gene expression from the *pel* operon promoter in response to c-di-GMP. Nucleic acids research.

[15] Nie H (2017). FleN and FleQ play a synergistic role in regulating *lapA* and *bcs* operons in *Pseudomonas putida* KT2440. Environmental microbiology reports.

[16] Baraquet C, Harwood CS (2013). Cyclic diguanosine monophosphate represses bacterial flagella synthesis by interacting with the Walker A motif of the enhancer-binding protein FleQ. Proceedings of the National Academy of Sciences of the United States of America.

[17] Molina-Henares MA, Ramos-Gonzalez MI, Daddaoua A, Fernandez-Escamilla AM, Espinosa-Urgel M (2017). FleQ of *Pseudomonas putida* KT2440 is a multimeric cyclic diguanylate binding protein that differentially regulates expression of biofilm matrix components. Res Microbiol.

[18] Matsuyama BY (2016). Mechanistic insights into c-di-GMP-dependent control of the biofilm regulator FleQ from *Pseudomonas aeruginosa*. Proceedings of the National Academy of Sciences of the United States of America.

[19] Matilla MA, Travieso ML, Ramos JL, Ramos-Gonzalez MI (2011). Cyclic diguanylate turnover mediated by the sole GGDEF/EAL response regulator in *Pseudomonas putida*: its role in the rhizosphere and an analysis of its target processes. Environmental microbiology.

[20] Nielsen L, Li X, Halverson LJ (2011). Cell-cell and cell-surface interactions mediated by cellulose and a novel exopolysaccharide contribute to *Pseudomonas putida* biofilm formation and fitness under water-limiting conditions. Environmental microbiology.

[21] Nilsson M (2011). Influence of putative exopolysaccharide genes on *Pseudomonas putida* KT2440 biofilm stability. Environmental microbiology.

[22] Xiao Y (2016). C-di-GMP regulates the expression of *lapA* and *bcs* operons via FleQ in *Pseudomonas putida* KT2440. Environmental microbiology reports.

[23] Baraquet C, Harwood CS (2016). FleQ DNA binding consensus sequence revealed by studies of FleQ-dependent regulation of biofilm gene expression in *Pseudomonas aeruginosa*. Journal of bacteriology.

[24] Martínez-Gil M, Ramos-González MI, Espinosa-Urgel M (2014). Roles of cyclic di-GMP and the Gac system in transcriptional control of the genes coding for the *Pseudomonas putida* adhesins LapA and LapF. Journal of bacteriology.

[25] Yousef-Coronado F, Travieso ML, Espinosa-Urgel M (2008). Different, overlapping mechanisms for colonization of abiotic and plant surfaces by *Pseudomonas putida*. FEMS Microbiol Lett.

[26] Ramos-Gonzalez MI (2016). Genetic dissection of the regulatory network associated with high c-di-GMP levels in *Pseudomonas putida* KT2440. Front Microbiol.

[27] Martínez-Granero F (2012). The Gac-Rsm and SadB signal transduction pathways converge on AlgU to downregulate motility in *Pseudomonas fluorescens*. PLoS One.

[28] Martínez-Granero F, Redondo-Nieto M, Vesga P, Martín M, Rivilla R (2014). AmrZ is a global transcriptional regulator implicated in iron uptake and environmental adaption in *P. fluorescens* F113. BMC genomics.

[29] Jones CJ (2014). ChIP-Seq and RNA-Seq reveal an AmrZ-mediated mechanism for cyclic di-GMP synthesis and biofilm development by *Pseudomonas aeruginosa*. PLoS pathogens.

[30] Muriel C (2018). AmrZ is a major determinant of c-di-GMP levels in *Pseudomonas fluorescens* F113. Sci Rep.

[31] Tart AH, Wolfgang MC, Wozniak DJ (2005). The alternative sigma factor AlgT represses *Pseudomonas aeruginosa* flagellum biosynthesis by inhibiting expression of *fleQ*. Journal of bacteriology.

[32] Martínez-Granero F (2014). Identification of *flgZ* as a flagellar gene encoding a PilZ domain protein that regulates swimming motility and biofilm formation in *Pseudomonas*. PLoS One.

[33] Duque, E. *et al*. In *Pseudomonas* Vol. V (ed. Ramos, J. L. and Filloux, A.) 227–254 (Springer, London, 2007).

[34] Kim JS, Kim YH, Anderson AJ, Kim YC (2014). The sensor kinase GacS negatively regulates flagellar formation and motility in a biocontrol bacterium, *Pseudomonas chlororaphis* O6. The plant pathology journal.

[35] Nogales J (2015). FleQ coordinates flagellum-dependent and -independent motilities in *Pseudomonas syringae* pv. tomato DC3000. Applied and environmental microbiology.

[36] Mastropaolo MD, Silby MW, Nicoll JS, Levy SB (2012). Novel genes involved in Pseudomonas fluorescens Pf0-1 motility and biofilm formation. Applied and environmental microbiology.

[37] Taylor TB (2015). Evolution. Evolutionary resurrection of flagellar motility via rewiring of the nitrogen regulation system. Science.

[38] Garrido-Sanz D (2016). Genomic and Genetic Diversity within the Pseudomonas fluorescens Complex. PLoS One.

[39] Shanahan P, O’Sullivan DJ, Simpson P, Glennon JD, O’Gara F (1992). Isolation of 2,4-diacetylphloroglucinol from a fluorescent pseudomonad and investigation of physiological parameters influencing its production. Applied and environmental microbiology.

[40] Schäfer A (1994). Small mobilizable multi-purpose cloning vectors derived from the *Escherichia coli* plasmids pK18 and pK19: selection of defined deletions in the chromosome of *Corynebacterium glutamicum*. Gene.

[41] Nakazawa T (2002). Travels of a *Pseudomonas*, from Japan around the world. Environmental microbiology.

[42] Bertani G (1951). Studies on lysogenesis. I. The mode of phage liberation by lysogenic *Escherichia coli*. Journal of bacteriology.

[43] Llamas MA, Bitter W (2006). Iron gate: the translocation system. Journal of bacteriology.

[44] de Lorenzo V, Eltis L, Kessler B, Timmis KN (1993). Analysis of *Pseudomonas* gene products using *lacIq/Ptrp-lac* plasmids and transposons that confer conditional phenotypes. Gene.

[45] Bolger AM, Lohse M, Usadel B (2014). Trimmomatic: a flexible trimmer for Illumina sequence data. Bioinformatics.

[46] Langmead B, Trapnell C, Pop M, Salzberg SL (2009). Ultrafast and memory-efficient alignment of short DNA sequences to the human genome. Genome biology.

[47] Li H (2009). The Sequence Alignment/Map format and SAMtools. Bioinformatics.

[48] Wysoker, A. T., K., Fennell, T. Picard. Sourceforge. net. http://picard.sourceforge.net, Accessed20 April 2016 (2013).

[49] Zhang Y (2008). Model-based analysis of ChIP-Seq (MACS). Genome biology.

[50] Rutherford K (2000). Artemis: sequence visualization and annotation. Bioinformatics.

[51] Ashburner M (2000). Gene ontology: tool for the unification of biology. The Gene Ontology Consortium. Nature genetics.

[52] Bailey TL (2009). MEME SUITE: tools for motif discovery and searching. Nucleic acids research.

[53] Altschul SF, Gish W, Miller W, Myers EW, Lipman DJ (1990). Basic local alignment search tool. Journal of molecular biology.

[54] Livak KJ, Schmittgen TD (2001). Analysis of relative gene expression data using real-time quantitative PCR and the 2(-Delta Delta C(T)) Method. Methods.

[55] Fox, J. *et al*. *Package ‘Rcmdr’* (2018).

[56] Chen H, Boutros PC (2011). VennDiagram: a package for the generation of highly-customizable Venn and Euler diagrams in R. BMC Bioinformatics.

[57] Team, R. C. R: *A language and environment for statistical computing*. (2013).

