# Supplementary material for: Genome-wide analysis of the FleQ direct regulon in Pseudomonas fluorescens F113 and Pseudomonas putida KT2440
Source: Sci Rep. 2018 Sep 3;8:13145. doi: 10.1038/s41598-018-31371-z (PMC6120874; doi:10.1038/s41598-018-31371-z)
Supplement: Supplementary file 1 — Supplementary Information [file 41598_2018_31371_MOESM1_ESM.pdf]

SUPPLEMENTARY INFORMATION

Genome-wide analysis of the FleQ direct regulon in *Pseudomonas fluorescens* F113 and *Pseudomonas putida* KT2440

Esther Blanco-Romero[1], Miguel Redondo-Nieto[1], Francisco Martínez-Granero[1], Daniel Garrido-Sanz[1], Maria Isabel Ramos-González[2], Marta Martín[1] and Rafael Rivilla[1*]

[1]Departamento de Biología, Facultad de Ciencias. Universidad Autónoma de Madrid. Darwin, 2. 28049. Madrid, Spain.

[2]Departamento de Protección Ambiental. Grupo de Microbiología Ambiental y Biodegradación. Estación Experimental del Zaidín, CSIC. Profesor Albareda, 1. 18008. Granada, Spain.

* Corresponding author: rafael.rivilla@uam.es

Supplementary Table 1. 159 genes putatively regulated by FleQ in *Pseudomonas fluorescens* F113. Genes resulting from ChIP-seq analysis of *Pseudomonas fluorescens* F113 and likely regulated by FleQ with a fold enrichment value equal or higher than five were sorted out. Those genes were compared with a Gene Ontology database to divide them into functional classes. The table includes the locus, gene and protein name and two statistical values: fold enrichment and –log10 (q-value) that indicate the probability of certainty of a binding site to be found. q-value = False discovery rate (FDR).

LOCUS	GENE	PRODUCT	FOLD ENRICHMENT	-log10(qvalue)
**c-di-GMP**				
PSF113_1630	-	GGDEF domain protein	10.24702	151.80345
PSF113_3487	-	Diguanylate phosphodiesterase	6.20649	55.60576
PSF113_4023	-	Diguanylate cyclase phosphodiesterase with PAS/PAC sensor	7.35137	77.72911
PSF113_5478	-	Response regulator sensory box GGDEF domain EAL domain-containing protein	7.60782	101.47101
PSF113_5738	-	GGDEF domain/EAL domain protein	6.88247	75.59928
**CELL WALL**				
PSF113_0208	*lapA*	LapA	7.82294	130.38884
PSF113_0453a	-	Putative glutamate racemase	6.10788	77.20217
PSF113_1539	-	Glucose-1-phosphate cytidylyltransferase	8.57087	98.20373
PSF113_1644	*wzz*	Wzz	5.51185	55.31474
PSF113_1970	-	UDP-glucose 6-dehydrogenase	5.96021	51.58108
PSF113_4136	-	Putative lipoprotein	8.12755	89.71924
PSF113_4182	*tadB*	TadB	5.66466	46.87431
PSF113_4752	*algD*	GDP-mannose 6-dehydrogenase	5.32818	51.14257
PSF113_5692	-	Putative lipoprotein	7.96255	115.96731
**IRON**				
PSF113_0933	*fagA*	FagA	11.28006	154.12726
PSF113_1274	-	TonB-dependent hemin, ferrichrome receptor	10.9213	184.4532
PSF113_1322	-	Iron-regulated protein A precursor	9.17467	169.18076
PSF113_1749	*pvdS*	PvdS	13.91936	215.40543
PSF113_1750	*pvdL*	PvdL	13.91936	215.40543
PSF113_1837	*pvdD*	PvdD	6.15185	60.2723
PSF113_1856	-	Outer membrane pyoverdine efflux protein	9.37042	151.79782
PSF113_1867	-	RNA polymerase sigma-70 factor, ECF subfamily	5.6154	46.10264
PSF113_2258	-	Outer membrane ferripyoverdine receptor	12.7578	187.10435
PSF113_2454	-	RNA polymerase sigma-70 factor, ECF subfamily	10.54119	138.24434
PSF113_2456	-	Ferrichrome-iron receptor	7.97978	86.94064
PSF113_2589	-	Ferrichrome-iron receptor	14.18627	217.55165
PSF113_2837	-	RNA polymerase sigma-70 factor, ECF subfamily	8.22607	91.58579
PSF113_3151	-	Ferrichrome-iron receptor	13.05476	213.74475
PSF113_3153	-	RNA polymerase sigma-70 factor, ECF subfamily	9.80232	122.81388
PSF113_3220	-	Heme uptake regulator	11.50129	175.22266
PSF113_3734	-	Ferrichrome-iron receptor	13.64444	206.80299
PSF113_4045	-	Iron-regulated membrane protein	11.13229	150.91508
PSF113_4536	-	Putative thiol oxidoreductase with 2 cytochrome c heme-binding sites	6.45278	59.71611
PSF113_4568	-	Bacterioferritin-associated ferredoxin	10.24495	185.12396
PSF113_4845	-	RNA polymerase sigma-70 factor, ECF subfamily	9.84721	149.09775
PSF113_4896	-	Ferrichrome-iron receptor	6.10524	62.40235

PSF113_5411	*fiuR*	FiuR	11.25993	191.21126
PSF113_5412	*fiuA*	FiuA	11.25993	191.21126
PSF113_5657	*fbpA*	FbpA	10.77871	177.21391
PSF113_5691	-	Cytochrome c family protein	7.96255	115.96731
**MOTILITY/CHEMOTAXIS**				
PSF113_0569	-	Methyl-accepting chemotaxis protein	11.50589	176.59119
PSF113_0751	*flhD*	FlhD	5.66387	63.05872
PSF113_1531	*flgF*	FlgF	11.92041	168.23859
PSF113_1532	*flgG*	FlgG	6.60056	62.22322
PSF113_1554	*fliC*	FliC	9.3253	121.64807
PSF113_1559	*fleQ*	FleQ	5.7599	107.76616
PSF113_1562	*fliE*	FliE	12.52218	197.44472
PSF113_1572	*fliL*	FliL	12.12493	176.80609
PSF113_1582	*flhA*	FlhA	10.32028	141.95418
PSF113_1583	*flhF*	FlhF	11.05789	151.57901
PSF113_2159	-	Methyl-accepting chemotaxis protein	7.96914	87.37503
PSF113_4454	*flgB*	FlgB	9.11271	108.85229
PSF113_4456	*cheV*	CheV	12.80387	190.9902
PSF113_4457	*flgA*	FlgA	12.80387	190.9902
PSF113_5017	-	Methyl-accepting chemotaxis protein	6.91812	85.27202
**OTHERS**				
PSF113_0079b	-	Putative restriction endonuclease	5.50442	73.33437
PSF113_0079c	-	Phage-related replication protein-like protein	8.62352	143.85052
PSF113_0159	-	NADH:ubiquinone oxidoreductase subunit 2 (chain N)	12.15716	205.77713
PSF113_0217	-	Cell division inhibitor	5.58049	50.11736
PSF113_0351	-	*yibQ* gene product, Possible divergent polysaccharide deacetylase	7.56204	83.46326
PSF113_0572	-	3'-to-5' exoribonuclease Rnase R	5.23404	47.34429
PSF113_0711	-	Exodeoxyribonuclease V gamma chain	11.42656	198.08304
PSF113_0869	*nusA*	NusA	6.19454	61.92004
PSF113_0878	*nadC*	NadC	5.49574	51.85266
PSF113_0889	-	Flavodoxin nitric oxide synthase	10.09099	152.12866
PSF113_1047	-	Multicopper oxidase	9.21123	110.81956
PSF113_1201	-	Ferredoxin--NADP(+) reductase	5.93565	59.21268
PSF113_1425	*purC*	PurC	6.81108	81.88702
PSF113_1491	-	Transposase-like protein	7.39861	103.25809
PSF113_1511	-	Putative hemagglutinin/hemolysin-related protein	7.23364	85.94704
PSF113_1592	*parA*	ParA	8.4231	95.35213
PSF113_1631	-	Dehydrogenases with different specificities (related to short-chain alcohol dehydrogenases)	10.24702	151.80345
PSF113_1652	-	Putative zinc-binding dehydrogenase	5.07468	43.46364
PSF113_1815	-	Integral membrane protein	12.64158	209.52077
PSF113_1971	-	Integral membrane protein	5.96021	51.58108
PSF113_2103	-	Phenylpropionate dioxygenase-related ring-hydroxylating dioxygenase, large terminal subunit	5.44679	47.21799
PSF113_2126	-	Dihydrodipicolinate synthase	10.23049	136.82835
PSF113_2158	*nuoA*	NuoA	7.96914	87.37503
PSF113_2455	-	Multidrug resistance protein B	7.97978	86.94064
PSF113_2559		UDP-N-acetylglucosamine 2-epimerase	5.12282	40.60619
PSF113_2611	*pdtF*	PdtF	5.36911	44.32938
PSF113_2636	-	Multidrug RND efflux membrane fusion protein	9.85158	123.8279

PSF113_2820	-	Peptidoglycan-binding domain 1 precursor	7.29017	74.29537
PSF113_2972	-	Glycosaminoglycan degradation	10.09787	128.92924
PSF113_3156	-	Dihydrofolate reductase	9.80232	122.81388
PSF113_3504	-	Transposase IS3/IS911 family protein	8.1647	91.08239
PSF113_3571	*phnB*	PhnB	9.11271	108.85229
PSF113_3889	-	Zinc carboxypeptidase domain protein	8.57087	98.20373
PSF113_3918	*tig*	Tig	6.60056	62.22322
PSF113_3922	*folD*	FolD	6.60056	62.22322
PSF113_4040	*pyrD*	PyrD	5.81243	49.21196
PSF113_4083	-	Sterol desaturase	8.22607	91.58579
PSF113_4204	-	Protein binding	11.08303	149.84854
PSF113_4394	-	Imidazoleglycerol-phosphate synthase	5.02416	40.56332
PSF113_4478	*rsmA*	RsmA	7.29191	88.92688
PSF113_4523	-	Mercuric reductase	5.89907	60.52337
PSF113_4567	-	Alkyl hydroperoxide reductase subunit C-like protein	10.24495	185.12396
PSF113_4932	*prs*	Prs	8.09361	100.50684
PSF113_4961	-	Paraquat-inducible protein A	11.39243	198.36824
PSF113_4978	-	Pentapeptide repeat-containing protein	5.31985	43.57669
PSF113_5275	*rpmJ*	RpmJ	5.41409	54.19064
PSF113_5294	*rplW*	RplW	6.1098	62.86637
PSF113_5307	*nusG*	NusG	9.25094	157.41789
PSF113_5315	*birA*	BirA	11.39243	198.36824
PSF113_5479	-	Alginate lyase precursor	7.60782	101.47101
PSF113_5482	*fda*	Fda	9.92493	158.95903
PSF113_5626	*spuA*	SpuA	7.29607	92.3802
PSF113_5627	*spuI*	SpuI	7.29607	92.3802
PSF113_5739	*rep*	Rep	6.88247	75.59928
PSF113_5765	*crc*	Crc	5.51407	52.63711
**REGULATION/SIGNAL TRANSDUCTION**				
PSF113_1200	-	LysR family transcriptional regulator	5.93565	59.21268
PSF113_1897	-	Transcriptional regulator, TetR family	8.32458	93.46353
PSF113_2274	-	Response regulator receiver domain protein	6.14595	64.4661
PSF113_3573	-	LysR family transcriptional regulator	5.22134	42.08332
PSF113_4024	-	Transcriptional regulator, Cro/CI family	7.35137	77.72911
PSF113_4373	-	DNA recombination-dependent growth factor C	5.31985	43.57669
PSF113_4470	*amrZ*	AmrZ	6.30233	70.2249
PSF113_4852	-	Transcriptional regulator, GntR family domain/Aspartate aminotransferase	5.15557	54.66805
**TRANSPORT**				
PSF113_0209	-	Type I secretion outer membrane family	7.82294	130.38884
PSF113_0210	-	Type I secretion system ATPase	5.71328	59.98046
PSF113_0350	-	ABC-type amino acid transport, signal transduction systems, periplasmic component/domain protein	7.56204	83.46326
PSF113_1510	-	Family type I secretion outer membrane protein	7.23364	85.94704
PSF113_1945	-	Permeases of the major facilitator superfamily	9.38239	116.90057
PSF113_1972	*attE*	AttE	7.93052	86.01953
PSF113_2040	-	Sugar ABC transporter (ATP-binding protein)	5.73097	49.77616
PSF113_2939	-	Amino acid transporter	6.45278	59.71611
PSF113_3572	-	Oligopeptide transporter, OPT family	5.22134	42.08332
PSF113_4178	*rcpA*	RcpA/CpaC	8.17681	90.65121

PSF113_4199	-	Transporter, LysE family	11.08303	149.84854
PSF113_4376	-	1-Acyl-sn-glycerol-3-phosphate acyltransferase	6.05872	53.18029
PSF113_4522	*aroP*	AroP	5.89907	60.52337
PSF113_4853		Benzoate transport protein	5.15557	54.66805
PSF113_5202	-	s-Methylmethionine permease	7.61864	98.55576
PSF113_5766	*gltS*	GltS	5.51407	52.63711
**UNKNOWN**				
PSF113_0216	-	Hypothetical protein	5.58049	50.11736
PSF113_0799	-	Hypothetical protein	5.70017	63.43042
PSF113_0800		Transmembrane protein	5.42595	56.96993
PSF113_0879		Thymidine phosphorylase	5.49574	51.85266
PSF113_1427	-	Hypothetical protein	6.81108	81.88702
PSF113_1581	-	Hypothetical protein	10.32028	141.95418
PSF113_1648	-	Hypothetical protein	6.26178	65.52363
PSF113_1944	-	Transmembrane protein	9.38239	116.90057
PSF113_2272	-	Hypothetical protein	5.84001	50.23604
PSF113_2273	-	Reticulocyte binding protein	5.84001	50.23604
PSF113_2819	-	Putative exported protein	7.29017	74.29537
PSF113_2938	-	Putative membrane protein	6.45278	59.71611
PSF113_4041	-	Hypothetical protein	5.81243	49.21196
PSF113_4392	-	YD repeat protein	6.83678	75.65234
PSF113_4468	-	Hemolysin activator protein precursor	6.30233	70.2249
PSF113_4474	-	Hypothetical protein	7.29191	88.92688
PSF113_4841	-	Hypothetical protein	7.83201	84.18573
PSF113_5053	-	Hypothetical protein	7.84626	104.39041
PSF113_5316	-	Hypothetical protein	11.39243	198.36824
PSF113_5481	-	Hypothetical protein	9.92493	158.95903
**VIRULENCE**				
PSF113_0324	-	Putative repressor protein from prophage	6.40209	76.4463
PSF113_0435	-	N-acetylglucosamine-binding protein A	5.73028	70.12623
PSF113_1855	-	RHS repeat-associated core domain-containing protein	9.37042	151.79782
PSF113_2409	*vgrG*	VgrG	7.97978	86.94064
PSF113_5816	*stp1*	Stp1	5.24984	55.61505

Supplementary Table 2. 160 genes putatively regulated by FleQ in *Pseudomonas putida* KT2440. Genes putatively regulated by FleQ obtained in ChIP-seq analysis from *Pseudomonas putida* KT2440 and presenting a fold enrichment value equal or higher than five were selected. Genes were then compared with a Gene Ontology database in order to sort them by functional classes. The table includes the locus, gene and protein name and two statistical values: fold enrichment and –log10 (q-value) that indicate the probability of certainty of a binding site to be found. q-value = False discovery rate (FDR).

LOCUS	GENE	PRODUCT	FOLD ENRICHMENT	-log10(qvalue)
**c-di-GMP**				
PP_0131	-	EAL domain containing protein	5	422.5
PP_0563	-	Responde regulator/GGDEF domain-containing protein	5.25	805.87
PP_5263	-	GGDEF/EAL domains containing protein	7.45	1022.86
**CELL WALL**				
PP_0116	*olpA*	Lipoprotein	10.04	3100
PP_0168	*lapA*	Surface adhesion protein	7.94	3100
PP_0504	*oprG*	OmpW family protein	5.64	1613.14
PP_1288	*algD*	GDP-mannose 6-dehydrogenase	7.36	833.37
PP_1778	-	Lipopolysaccharide ABC export system, permease	6.26	311.39
PP_1795		Polysaccharide synthesis, Peb	5.96	414.67
PP_1970	-	Lipoprotein	11.77	2113.79
PP_2156	*lolE*	Lipoprotein releasing system transmembrane protein LolC/E family	5.22	252.28
PP_2191	-	Lipoprotein	11.46	1891.76
PP_2629	-	Pseudogene (first in Bcs operon)	5.46	417.14
PP_3126	-	Polysaccharide export protein, Pea	7.41	1044.62
PP_3733	-	ABC transporter	7.88	805.62
PP_4057	-	Outer membrane autotransporter	6.92	846.73
**IRON**				
PP_0160	-	TonB-dependent siderophore receptor	8.15	3100
PP_0162	-	ECF family RNA polymerase sigma-70 factor	8.72	2000.61
PP_0180	-	Cytochrome c family protein	8.62	1134.96
PP_0267	-	Ferric siderophore receptor	8.45	1817.23
PP_0350	-	Ferrichrome-iron receptor	5.1	270.43
PP_0352	-	ECF family RNA polymerase sigma-70 factor	5.14	376.97
PP_0535	-	TonB-dependent siderophore receptor	8.34	1196.04
PP_0667	-	ECF family RNA polymerase sigma-70 factor	6.89	985.6
PP_0704	-	ECF family RNA polymerase sigma-70 factor	8.19	955.21
PP_0861	-	Outer membrane ferric siderophore receptor	9.91	2226.12
PP_1006	-	Heme receptor	9.91	1259.64
PP_1082	*bfr*	Bacterioferritin	10.92	3100
PP_1083	-	BFD(2Fe-2S)-binding domain-containing protein	10.92	3100
PP_2192	-	ECF family RNA polymerase sigma-70 factor/transmembrane sensor protein	11.46	1891.76
PP_2590	-	Outer membrane ferric siderophore receptor	13.26	3100
PP_3086	-	ECF family RNA polymerase sigma-70 factor	9.12	1284.71
PP_3325	-	Outer membrane ferric siderophore receptor	8.73	856.6
PP_3612	-	TonB-dependent siderophore receptor	5.77	314.42
PP_4606	-	TonB-dependent siderophore receptor	5	198.67
PP_4611	-	ECF family RNA polymerase sigma-70 factor	13.24	3100

PP_4755	-	TonB-dependent siderophore receptor	10.97	3100
PP_4881	-	Iron ABC transporter substrate-binding protein	6.12	996.08
PP_5306	*exbB*	Ferric siderophore transport system protein ExbB	10.11	3112.64
**MOTILITY/CHEMOTAXIS**				
PP_0952	*rpoN*	RNA polymerase factor sigma-54	5.07	293.21
PP_2310	-	Methyl-accepting chemotaxis sensory transducer	6.71	363.45
PP_4335	*motB*	Flagellar motor protein MotD	13.8	3100
PP_4343	*flhF*	Flagellar biosynthesis regulator FlhF	11.96	3100
PP_4344	*flhA*	Flagellar biosynthesis protein FlhA	11.96	3100
PP_4352	*flhB*	Flagellar biosynthesis protein FlhB	5.97	261.34
PP_4361	*fliK*	Flagellar hook-length control protein	12.88	3100
PP_4370	*fliE*	Flagellar hook-basal body protein FliE	7.13	2512.84
PP_4385	*flgG*	Flagellar basal body rod protein FlgG	12	3100
PP_4386	*flgF*	Flagellar basal body rod protein FlgF	12	3100
PP_4391	*flgB*	Flagellar basal-body rod protein FlgB	11.19	3100
PP_4393	*cheV-3*	Chemotaxis protein CheV	11.19	3100
PP_4394	*flgA*	Flagellar basal body P-ring biosynthesis protein FlgA	11.19	3100
PP_4395	*flgM*	Anti-sigma-28 factor FlgM	11.19	3100
PP_4888	-	Methyl-accepting chemotaxis sensory transducer	8.75	923.85
**OTHERS**				
PP_0117	*znuB*	Hypothetical protein	10.04	3100
PP_0159	-	CAIB/BAIF family protein	8.15	3100
PP_0420	*trpG*	Anthranilate synthase component II	7.27	805.47
PP_0437	*birA*	Biotin-protein ligase	7.21	3100
PP_0440	*tuf*	Elongation factor Tu	5.07	806.87
PP_0545	*aldB-I*	Aldehyde dehydrogenase	7.98	800.42
PP_0625	*clpB*	ATP-dependent Clp protease, ATP-binding subunit ClpB	6.69	1288.56
PP_0626	*ndh*	FAD-dependent pyridine nucleotide-disulfide oxidoreductase	6.69	1288.56
PP_0705	*alkA*	DNA-3-methyladenine glycosylase II	8.19	955.21
PP_0787	*nadC*	Nicotinate-nucleotide pyrophosphorylase	6.47	517.55
PP_0788	-	Hypothetical protein	6.47	517.55
PP_1003	*arcD-II*	Arginine/ornithine antiporter	7.39	3222.65
PP_1084	-	Anti-oxidant AhpCTSA family protein	10.92	3100
PP_1085	*rnt*	Ribonuclease T	10.92	3100
PP_1184	-	Dienelactone hydrolase	6.24	2137.62
PP_1207	-	Histidine triad (HIT)	9.06	3100
PP_1638	*fpr*	Oxidoreductase FAD/NAD(P)-binding domain-containing protein	5.93	1930.2
PP_1777	*xanA*	Phosphomannomutase	6.26	311.39
PP_1785	*rmlB*	dTDP-glucose 4,6-dehydratase	6.73	556.37
PP_1786	-	Glycosyl transferase family protein	6.73	556.37
PP_1878	-	Hypothetical protein	5.69	1202.2
PP_2220	-	C4-type zinc finger DksA/TraR family protein	13.18	1816.25
PP_2239	*rhtA*	Cysteine transporter	8.6	3100
PP_2736	-	Amine oxidase	5.07	158.32
PP_2737	-	Short chain dehydrogenase/reductase oxidoreductase	5.07	158.32
PP_3184	*mcoA*	Mn(II) copper oxidase A	5.07	234.84
PP_3324	-	GTP cyclohydrolase	8.73	856.6

PP_3547	-	Short chain dehydrogenase/reductase oxidoreductase	6.86	214.19
PP_3569	*quiA*	Quinate dehydrogenase	6.32	310.87
PP_3700	*parI*	Chromosome partitioning ATPase	11.8	3100
PP_3794	*pbpG*	D-alanyl-D-alanine endopeptidase	6.83	885.21
PP_3839	*adhP*	Alcohol dehydrogenase	5.35	876.29
PP_4070	*panC*	Pantothenate synthetase	7.25	706.37
PP_4316	-	2-hydroxyacid dehydrogenase	14.42	3100
PP_4334	-	ParA family protein	13.8	3100
PP_4350	-	Aminotransferase	5.97	261.34
PP_4469	*gmk-1*	Phosphonate metabolism protein/1,5-bisphosphokinase PhnN	5.41	558.29
PP_4674	*recC*	Exodeoxyribonuclease V subunit gamma	7.67	3100
PP_4697	*pcnB*	Poly(A) polymerase	6.34	702.14
PP_4880	*vacB*	Ribonuclease R	6.12	996.08
PP_4960	*fda*	Fructose-1,6-bisphospate aldolase	5.48	323.23
PP_5045	*thiI*	Thiamine biosynthesis protein ThiI	5.16	251.68
PP_5046	*glnA*	Glutamine synthetas, type I	5.16	251.68
PP_5059	-	Hypothetical protein	5.66	350.3
PP_5264	*rep*	ATP-dependent DNA helicase Rep	7.45	1022.86
PP_5305	-	NAD-dependet epimerase/dehydratase	10.11	3112.64
**REGULATION/SIGNAL TRANSDUCTION**				
PP_0546	-	Fis family transcriptional regulator	7.98	800.42
PP_1012	*gltR-2*	Winged helix family	6.08	270.68
PP_1013	-	Integral membrane sensor signal transduction histidine kinase	6.08	270.68
PP_1186	*phoP*	Winged helix family two component transcriptional regulator	6.24	2137.62
PP_1637	-	LysR family transcriptional regulator	5.93	1930.2
PP_1650	*gacS*	Multi-sensor hybrid histidine kinase	7.8	750.17
PP_1651	-	Two-component system response regulator	7.8	750.17
PP_1978	-	TetR family transcriptional regulator	6	1649.59
PP_3185	*pet18*	TenA family transcriptional activator	5.07	234.84
PP_3503	-	Fis family transcriptional regulator	6	505.75
PP_4345	-	GntR family transcriptional regulator	11.96	3100
PP_4470	*amrZ*	Arc domain-contaning protein DNA binding domain-containing protein	5.41	558.29
**TRANSPORT**				
PP_0167	-	LapA type I secretion system ATPase	7.94	3100
PP_0268	-	Porin	8.45	1817.23
PP_1002	*arcD*	Arginine/ornithine antiporter	7.39	3222.65
PP_1150	-	Fusaric acid resistance protein	6.11	980.7
PP_1156	-	Formate/nitrate transporter	7.33	3100
PP_1185	*oprH*	Outer membrane protein H1	6.24	2137.62
PP_1206	*oprD*	Porin	9.06	3100
PP_1728	-	Hypothetical protein	8.5	771.6
PP_1797	-	HlyD family secretion protein	5.96	414.67
PP_2240	-	ABC transporter	8.6	3100
PP_2429	-	Lysine exporter protein LysE/YggA	5.22	121.02
PP_2628	-	ABC transporter ATP-binding protein	5.46	417.14
PP_3789	-	Efflux transporter	6.74	548.54
PP_4458	-	Opine ABC transporter substrate-binding protein	6.93	1609.29

PP_4471	*mgtE*	Magnesium transporter	6.05	2418.49
PP_4519	*tolC*	TolC-type I secretion outer membrane protein	7.77	2603.71
PP_5206	*yhiI*	Secretion protein HlyD family protein	6.27	2140.15
**UNKNOWN**				
PP_0419	*estP*	Hypothetical protein	7.27	805.47
PP_0534	-	Winged helix family two component transcriptional regulator	8.34	1196.04
PP_0564	-	Hypothetical protein	5.25	805.87
PP_0599	-	Hypothetical protein	6.37	3100
PP_0681	-	Hypothetical protein	8.47	1461.39
PP_0685	-	Hypothetical protein	6.26	653.12
PP_0862	-	Hydroxylase	9.91	2226.12
PP_1148	-	Hypothetical protein	6.11	980.7
PP_1149	-	Hypothetical protein	6.11	980.7
PP_1157	-	Acetolactate synthase	7.33	3100
PP_1864	-	Hypothetical protein	13.88	2112.38
PP_1865	-	ISPpu8, transposase	13.88	2112.38
PP_1991	-	Hypothetical protein	10.6	937.02
PP_2114	-	ISPpu8, transposase	8.03	548.41
PP_2219	-	Hypothetical protein	13.18	1816.25
PP_2473	-	Hypothetical protein	6.24	713.26
PP_2509	-	Hypothetical protein	6.32	347.57
PP_3011	-	Hypothetical protein	6.94	583.83
PP_3101	-	ADP-ribosylglycohydrolase	5.07	195.17
PP_3504	-	Hypothetical protein	6	505.75
PP_3611	-	Hypothetical protein	5.77	314.42
PP_3685	-	Hypothetical protein	5.13	281.74
PP_3686	-	Hypothetical protein	6.51	766.17
PP_3696	-	Hypothetical protein	9.24	1142.42
PP_4317	-	Hypothetical protein	14.42	3100
PP_4318	-	ISPpu8, transposase	14.42	3100
PP_4360	-	Hypothetical protein	12.88	3100
PP_4414	-	Hypothetical protein	6.52	763.32
PP_4416	-	Hypothetical protein	6.52	763.32
PP_4459	-	Transposase	6.93	1609.29
PP_4610	-	PepSY-associated TM helix domain-containing protein	13.24	3100

Supplementary Table 3. 240 genes likely regulated by FleQ in *Pseudomonas fluorescens* F113 *bifA*$^-$. Genes putatively regulated by FleQ in *Pseudomonas fluorescens* F113 *bifA*$^-$ background obtained from ChIP-seq analysis with a fold enrichment value equal or higher than five were sorted out. Selected genes were functionaly classified according to Gene Ontology database. The table includes the locus, gene and protein name, two statistical values: fold enrichment and –log10 (q-value) that indicate the probability of certainty of a binding site to be found; and the comparison with *Pseudomonas fluorescens* F113 ChIP-seq and *Pseudomonas fluorescens* F113 sadC$^-$ wspR$^-$ ChIP-seq assays. q-value = False discovery rate (FDR).

LOCUS	GENE	PRODUCT	FOLD ENRICHMENT	-log10(qvalue)	GENE FOUND IN F113 ChIP-seq	GENE FOUND IN F113 sadC$^-$ wspR$^-$ ChIP-seq
**c-di-GMP**						
PSF113_1630	-	GGDEF domain protein	9.35	1235.18	Yes	No
PSF113_3487	-	Diguanylate phosphodiesterase	12.72	1667.49	Yes	Yes
PSF113_4023	-	Diguanylate cyclase phosphodiesterase with PAS PAC sensor	10.02	1137.94	Yes	Yes
PSF113_5478	-	Response regulator sensory box GGDEF domain EAL domain-containing protein	6.85	796.86	Yes	Yes
PSF113_5738	-	GGDEF domain/EAL domain protein	9.72	1332.3	Yes	Yes
**CELL WALL**						
PSF113_0208	*lapA*	LapA	8.47	1497.41	Yes	Yes
PSF113_1644	*wzz*	Wzz	10.96	1612.71	Yes	Yes
PSF113_1647	*matE*	MatE	9.51	1216.18	No	No
PSF113_1970	-	UDP-glucose 6-dehydrogenase	6.43	618.05	Yes	No
PSF113_3312	-	Putative lipoprotein	7.01	618.12	No	No
PSF113_4136	-	Putative lipoprotein	6.47	534.43	Yes	No
PSF113_4777	*LpxC*	LpxC	6.07	518.72	No	Yes
PSF113_5692	-	Putative lipoprotein	9.69	1579.61	Yes	No
**IRON**						
PSF113_0198	*foxI*	FoxI	8	990.18	No	Yes
PSF113_1274	-	TonB-dependent hemin , ferrichrome receptor	12.48	2210.13	Yes	Yes
PSF113_1322	-	Iron-regulated protein A precursor	5.71	1265.1	Yes	Yes
PSF113_1749	*pvdS*	PvdS	15.61	2492.19	Yes	Yes
PSF113_1837	*pvdD*	PvdD	12.91	1705.15	Yes	Yes
PSF113_1856	-	Outer membrane pyoverdine eflux protein	11.78	2113.91	Yes	Yes
PSF113_1867	-	RNA polymerase sigma-70 factor, ECF subfamily	10.43	1214.69	Yes	Yes
PSF113_1869	*fecA*	FecA	7.59	711.4	No	Yes
PSF113_2258	-	Outer membrane ferripyoverdine receptor	16.68	2522.55	Yes	Yes
PSF113_2454	-	RNA polymerase sigma-70 factor, ECF subfamily	15.96	2360.69	Yes	Yes
PSF113_2456	-	Ferrichrome-iron receptor	14.42	2023.83	Yes	Yes
PSF113_2589	-	Ferrichrome-iron receptor	16.72	2586.58	Yes	Yes
PSF113_2837	-	RNA polymerase sigma-70 factor, ECF subfamily	12.85	1694.05	Yes	Yes
PSF113_3151	-	Ferrichrome-iron receptor	14.76	2444.76	Yes	Yes
PSF113_3153	-	RNA polymerase sigma-70 factor, ECF subfamily	15.09	2169.43	Yes	Yes
PSF113_3220	-	Heme uptake regulator	15.74	2458.56	Yes	Yes
PSF113_3734	-	Ferrichrome-iron receptor	16.77	2543.16	Yes	Yes
PSF113_3792	-	Periplasmic protein p19 involved in high-affinity Fe2+ transport	5.61	409.48	No	Yes
PSF113_4045	-	Iron-regulated membrane protein	15.04	2158.86	Yes	Yes
PSF113_4536	-	Putative thiol oxidoreductase with 2 cytochrome c heme-binding sites	12.55	1632.25	Yes	Yes
PSF113_4568	-	Bacterioferritin-associated ferredoxin	9.97	1946.39	Yes	Yes
PSF113_4845	-	RNA polymerase sigma-70 factor, ECF subfamily	13.8	2249.3	Yes	Yes
PSF113_4896	-	Ferrichrome-iron receptor	12.27	1828.2	Yes	Yes
PSF113_4983	-	Periplasmic hemin-binding protein	5.74	470.3	No	Yes
PSF113_5410	*fiuI*	FiuI	7.74	901.75	No	Yes
PSF113_5412	*fiuA*	FiuA	13.35	2286.3	Yes	No
PSF113_5482	*fda*	Fda	10.69	1774.1	Yes	Yes
PSF113_5657	*fbpA*	FbpA	11.58	1996.71	Yes	Yes
PSF113_5672	-	TonB-like protein	5.43	687.92	No	Yes
PSF113_5691	-	Cytochrome c family protein	9.69	1579.61	Yes	Yes
**MOTILITY/CHEMOTAXIS**						
PSF113_0199	-	Methyl-accepting chemotaxis protein	8	990.18	No	Yes
PSF113_0569	-	Methyl-accepting chemotaxis protein	7.09	873.61	Yes	Yes
PSF113_1434	-	Methyl-accepting chemotaxis protein	8.08	894.42	No	Yes
PSF113_1531	*flgF*	FlgF	9.8	1097.09	Yes	No

PSF113_1562	*fliE*	FliE	6.44	1213.74	Yes	Yes
PSF113_1572	*fliL*	FliL	11.6	1440.78	Yes	Yes
PSF113_1579	*flhB*	FlhB	6.04	470.72	No	Yes
PSF113_1582	*flhA*	FlhA	8.83	992.96	Yes	No
PSF113_1582	*flhA*	FlhA	9.88	1124.31	Yes	No
PSF113_1583	*flhF*	FlhF	9.88	1124.31	Yes	No
PSF113_1591	*motB*	MotB	8.08	791.88	No	No
PSF113_2159	-	Methyl-accepting chemotaxis protein	11.2	1361.51	Yes	Yes
PSF113_2925	-	Methyl-accepting chemotaxis protein	5.31	368.2	No	Yes
PSF113_4454	*flgB*	FlgB	6.79	583.73	Yes	No
PSF113_4456	*cheV*	CheV	13.48	1982.66	Yes	Yes
PSF113_4457	*flgA*	FlgA	13.48	1982.66	Yes	Yes
PSF113_5017	-	Methyl-accepting chemotaxis protein	7.85	1208.12	Yes	Yes
**OTHERS**						
PSF113_0079b	-	Putative restriction endonuclease	6.09	1060.66	Yes	Yes
PSF113_0079c	-	Phage-related replication protein-like protein	9.96	1790.84	Yes	Yes
PSF113_0139	*trpA*	TrpA	7.16	870.4	No	Yes
PSF113_0159	-	NADH:ubiquinone oxidoreductase subunit 2 (chain N)	13.59	2229.66	Yes	Yes
PSF113_0217	-	Cell division inhibitor	6.98	701.92	Yes	No
PSF113_0351	-	YibQ gene product, possible divergent polysaccharide deacetylase	7.56	797.04	Yes	Yes
PSF113_0572	-	3'-to-5' exoribonuclease RNase R	6	541.44	Yes	Yes
PSF113_0624	*urtD*	UrtD	6.41	561.21	No	Yes
PSF113_0711	-	Exodeoxyribonuclease V gamma chain	14.58	2312.93	Yes	Yes
PSF113_0869	*nusA*	NusA	7.8	766.39	Yes	Yes
PSF113_0870	*infB*	InfB	7.15	759.87	No	Yes
PSF113_0889	-	Flavodoxin nitric oxide synthase	11.36	1891.01	Yes	Yes
PSF113_0934	-	Fumarate hydratase class II	12.41	1823.36	No	No
PSF113_0994	-	Lysogenic conversion protein	6.53	814.8	No	No
PSF113_1047	-	Multicopper oxidase	13.59	1847.6	Yes	Yes
PSF113_1069	*yaeQ*	YaeQ	7.73	811.32	No	Yes
PSF113_1201	-	Ferredoxin--NADP(+) reductase	8.77	1214.83	Yes	Yes
PSF113_1216	-	Methylated-DNA--protein-cysteine methyltransferase-related protein	10.11	1270.9	No	Yes
PSF113_1217	-	Peptide chain release factor RF-3	10.11	1270.9	No	Yes
PSF113_1491	-	Transposase-like protein	9.71	1821.23	Yes	Yes
PSF113_1511	-	Putative hemagglutinin/hemolysin-related protein	8.54	990.07	Yes	Yes
PSF113_1580	-	YD repeat protein	6.04	470.72	No	No
PSF113_1592	*parA*	ParA	8.08	791.88	Yes	No
PSF113_1657	-	Acyltransferase 3	9	1292.01	No	Yes
PSF113_1658	-	O-antigen acetylase	9	1292.01	No	Yes
PSF113_1815	-	Integral membrane protein	13.93	2254.69	Yes	Yes
PSF113_1836	-	Putative thioesterase involved in non-ribosomal peptide biosynthesis	12.91	1705.15	No	Yes
PSF113_1919	-	Oxidoreductase, short chain dehydrogenase/reductase family	5.04	332.5	No	Yes
PSF113_1923	-	FAD dependent oxidoreductase	5.04	332.5	No	Yes
PSF113_1971	-	Integral membrane protein	6.43	618.05	Yes	No
PSF113_2016	-	Threonyl-tRNA synthetase	5.46	388.67	No	No
PSF113_2058	*arsH*	ArsH	5.86	445.15	No	Yes
PSF113_2103	-	Phenylpropionate dioxygenase-related ring-hydroxylating dioxygenase, large terminal subunit	6.99	646.94	Yes	Yes
PSF113_2126	-	Dihydrodipicolinate synthase	12.04	1709.85	Yes	Yes
PSF113_2158	*nuoA*	NuoA	11.2	1361.51	Yes	Yes
PSF113_2455	-	Multidrug resistance protein B	14.42	2023.83	Yes	No
PSF113_2559	-	UDP-N-acetylglucosamine 2-epimerase	6.86	615.52	Yes	No
PSF113_2611	*pdtF*	PdtF	10.05	1141.95	Yes	Yes
PSF113_2636	-	Multidrug RND efflux membrane fusion protein	12.71	1664.18	Yes	Yes
PSF113_2646	*mutT*	MutT	5.88	448.23	No	Yes
PSF113_2698	*ligD*	LigD	7.4	680.77	No	Yes
PSF113_2820	-	Peptidoglycan-binding domain 1 precursor	5.36	374.74	Yes	No
PSF113_2926	-	Aminotransferase class I and II	5.31	368.2	No	Yes
PSF113_2972	-	Putative membrane protein	15.92	2353.49	Yes	Yes
PSF113_3156	-	Dihydrofolate reductase	15.09	2169.43	Yes	Yes
PSF113_3162	-	Dienelactone hydrolase-related enzyme	8.59	879.36	No	Yes
PSF113_3293	-	Relaxase	5.47	459.01	No	No

PSF113_3307	-	Acetate kinase	5.15	347.35	No	Yes
PSF113_3308	-	Rossmann fold nucleotide-binding domain-containing protein	5.15	347.35	No	Yes
PSF113_3422	*nosR2*	NosR2	7.19	646.23	No	Yes
PSF113_3423	-	Nitrous-oxide reductase	7.19	646.23	No	No
PSF113_3423	-	Nitrous-oxide reductase	6.61	578.56	No	No
PSF113_3424	*nosF2*	NosD2	6.61	578.56	No	No
PSF113_3485	-	Aldehyde dehydrogenase	5.64	413.24	No	No
PSF113_3486	*ropAA-2*	RopAA-2	5.64	413.24	No	No
PSF113_3504	-	Transposase is3 is911 family protein	6.39	557.99	Yes	Yes
PSF113_3571	*phnB*	PhnB	13.76	1882.8	Yes	Yes
PSF113_3785	-	Ribonucleotide reductase of class III (anaerobic), large subunit	5.01	328.99	No	Yes
PSF113_3795	-	Putative collagenase	5.8	435.95	No	Yes
PSF113_3889	-	Zinc carboxypeptidase domain protein	13.82	1896.46	Yes	Yes
PSF113_3918	*tig*	Tig	9.87	1109.01	Yes	Yes
PSF113_3922	*folD*	FolD	9.87	1109.01	Yes	Yes
PSF113_4083	-	Sterol desaturase	14.48	2036.56	Yes	Yes
PSF113_4204	-	Protein binding	12.01	1523.33	Yes	Yes
PSF113_4280	-	DNA mismatch repair protein	6.73	883.28	No	Yes
PSF113_4393	-	Imidazoleglycerol-phosphate synthase	11.96	1876.17	No	Yes
PSF113_4394	-	Imidazoleglycerol-phosphate synthase	8.41	934.63	Yes	Yes
PSF113_4478	*rsmA*	RsmA	9.39	1210.6	Yes	Yes
PSF113_4523	-	Mercuric reductase	10.08	1403.74	Yes	Yes
PSF113_4751	*yaaA*	YaaA	6.18	645.68	No	No
PSF113_4804	*rplM*	RplM	5.61	439.38	No	No
PSF113_4805	-	Oxidoreductase, aldo/keto reductase family	5.61	439.38	No	No
PSF113_4848	-	Ribosomal subunit interface	6.17	527.26	No	Yes
PSF113_4932	*prs*	Prs	9.51	1343.49	Yes	Yes
PSF113_4960	-	Paraquat-inducible protein A	12.69	2174.63	No	No
PSF113_4979	-	Regulator of competence-specific genes	5	380.96	No	Yes
PSF113_4984	-	Rieske 2Fe-2S family protein	5.74	470.3	No	Yes
PSF113_4992	*pcnB*	PcnB	6.05	555.63	No	No
PSF113_5096	-	4-hydroxyphenylpyruvate dioxygenase	5.16	368.3	No	Yes
PSF113_5146	*leuS*	LeuS	8.8	1127.39	No	Yes
PSF113_5200	-	Threonine synthase	5.02	353.78	No	Yes
PSF113_5322	*tyrZ*	TyrZ	12.74	2187.83	No	Yes
PSF113_5389	-	Stress induced hydrophobic peptide	5.87	563.54	No	Yes
PSF113_5479	-	Alginate lyase precursor	6.85	796.86	Yes	Yes
PSF113_5626	*spuA*	SpuA	8.14	1219.43	Yes	Yes
PSF113_5627	*spuI*	SpuI	8.14	1219.43	Yes	Yes
PSF113_5727	-	ATPase involved in chromosome partitioning	7.56	1213.02	No	No
PSF113_5739	*rep*	Rep	9.72	1332.3	Yes	Yes
PSF113_5773	-	Putative translation initiation inhibitor, yjgF family	6.97	744.41	No	No
PSF113_5844	-	Homocysteine S-methyltransferase	7.38	980.85	No	Yes
**REGULATION/SIGNAL TRANSDUCTION**						
PSF113_0563	*hfq*	Hfq	8.24	1101.33	No	Yes
PSF113_0990a	-	Transcriptional regulator, TetR family	5.92	540.52	No	Yes
PSF113_1200	-	LysR family transcriptional regulator	8.77	1214.83	Yes	Yes
PSF113_1439	-	Transcriptional regulator, LysR family	5.47	425.85	No	Yes
PSF113_1719	-	DNA-binding response regulator, LuxR family	5.64	413.99	No	Yes
PSF113_1897	-	Transcriptional regulator, TetR family	7.96	772.88	Yes	Yes
PSF113_1927	-	Transcriptional regulator, AraC family	6.01	468.24	No	Yes
PSF113_2256	-	Transcriptional regulator	7.4	680.77	No	Yes
PSF113_2274	-	Response regulator receiver domain protein	10.49	1445.33	Yes	No
PSF113_2728	-	Transcriptional regulator, TetR family	5	326.89	No	Yes
PSF113_3264	-	Transcriptional regulator, IclR family	6.61	556.46	No	Yes
PSF113_3439	*pnpR*	PnpR	6.13	532.95	No	Yes
PSF113_3573	-	Transcriptional regulator, LysR family	5.66	416.25	Yes	No
PSF113_4024	-	Transcriptional regulator, Cro/CI family	10.02	1137.94	Yes	Yes
PSF113_4373	-	DNA recombination-dependent growth factor C	6.07	475.41	Yes	Yes
PSF113_4470	*amrZ*	AmrZ	6.56	706.08	Yes	No
PSF113_4792	*mraZ*	MraZ	6.38	643.58	No	Yes

PSF113_5199	-	Transcriptional regulator, AsnC family	5.02	353.78	No	No
PSF113_5280	*rplR*	RplR	9.97	1435.41	No	Yes
PSF113_5300	*rpsL*	RpsL	6.82	765.71	No	No
PSF113_5301	*rpoC*	RpoC	5.15	438.32	No	Yes
**TRANSPORT**						
PSF113_0209	-	Type I secretion outer membrane family	8.47	1497.41	Yes	Yes
PSF113_0350	-	ABC-type amino acid transport, signal transduction systems, periplasmic component/domain protein	7.56	797.04	Yes	No
PSF113_1510	-	Family type I secretion outer membrane protein	8.54	990.07	Yes	Yes
PSF113_1945	-	Permeases of the major facilitator superfamily	8.83	945.94	Yes	Yes
PSF113_1972	*attE*	AttE	6.96	609.69	Yes	Yes
PSF113_2040	-	Sugar ABC transporter (ATP-binding protein)	10.64	1253.55	Yes	Yes
PSF113_2560	-	TRAP-type transport system, small permease component, predicted N-acetylneuraminate transporter	6.86	615.52	No	No
PSF113_2676	*nhaB*	NhaB	6.44	529.56	No	Yes
PSF113_3572	-	Oligopeptide transporter, OPT family	5.66	416.25	Yes	No
PSF113_3993	-	ABC transporter, ATP-binding/permease protein	7.75	737.07	No	Yes
PSF113_4178	*rcpA*	RcpA/CpaC	7.99	777.39	Yes	Yes
PSF113_4199	-	Transporter, LysE family	12.01	1523.33	Yes	Yes
PSF113_4376	-	1-acyl-sn-glycerol-3-phosphate acyltransferase	9.66	1071.39	Yes	No
PSF113_4377	-	C4-dicarboxylate transporter/malic acid transport protein	9.66	1071.39	No	Yes
PSF113_4522	*aroP*	AroP	10.08	1403.74	Yes	Yes
PSF113_4581	*gltI*	GltI	5.79	519.61	No	Yes
PSF113_5202	-	S-methylmethionine permease	10.14	1221.83	Yes	No
PSF113_5308	*secE*	SecE	10.07	1898.35	No	Yes
PSF113_5673	*glpT*	GlpT	5.43	687.92	No	Yes
PSF113_5845	-	Amino acid ABC transporter, periplasmic amino acid-binding protein	7.38	980.85	No	Yes
**UNKNOWN**						
PSF113_0145	-	Hypothetical protein	5.43	642.72	No	Yes
PSF113_0216	-	Hypothetical protein	6.98	701.92	Yes	No
PSF113_0324	-	Hypothetical protein	9.03	1487.04	Yes	Yes
PSF113_0453b	-	Hypothetical protein	8.57	1544.7	Yes	No
PSF113_0799	-	Hypothetical protein	8.43	1293.8	Yes	Yes
PSF113_0800	-	Transmembrane protein	8.83	1253.16	Yes	Yes
PSF113_0879	-	Thymidine phosphorylase	6.47	630.95	Yes	No
PSF113_0928	-	Putative exported protein	7.42	683.38	No	Yes
PSF113_0990	-	4-oxalocrotonate tautomerase family protein	5.92	540.52	No	Yes
PSF113_1170	-	Dockerin type 1 protein	5.66	516.97	No	No
PSF113_1177	-	Prophage long tail fiber protein H	5.53	451.66	No	No
PSF113_1427	-	Hypothetical protein	10.51	1535.65	Yes	Yes
PSF113_1438	-	Hypothetical protein	5.47	425.85	No	Yes
PSF113_1581	-	Hypothetical protein	8.83	992.96	Yes	No
PSF113_1651	-	Glycosyl hydrolase bnr repeat-containing protein	8.22	977.03	No	Yes
PSF113_1656	*wbpL*	WbpL	5.78	510.04	No	Yes
PSF113_1937	-	Hypothetical protein	5.51	478.3	No	Yes
PSF113_1944	-	Transmembrane protein	8.83	945.94	Yes	Yes
PSF113_2265a	-	Putative integron gene cassette protein	6.09	583.52	No	No
PSF113_2266	-	Hypothetical protein	6.09	583.52	No	No
PSF113_2272	-	Hypothetical protein	8.06	833.42	Yes	Yes
PSF113_2273	-	Reticulocyte binding protein	10.49	1445.33	Yes	Yes
PSF113_2410	-	Hypothetical protein	12.24	1568.85	No	No
PSF113_2522	-	Hypothetical protein	7.5	696.47	No	Yes
PSF113_2645	-	Putative exported protein	5.88	448.23	No	Yes
PSF113_2819	-	Putative exported protein	5.36	374.74	Yes	No
PSF113_2938	-	Putative membrane protein	7.72	731.74	Yes	Yes
PSF113_2956	-	Hypothetical protein	8.34	835.76	No	Yes
PSF113_3171	-	Hypothetical protein	9.18	984.67	No	Yes
PSF113_3311	-	Putative membrane protein	7.01	618.12	No	Yes
PSF113_3793	-	Hypothetical protein	5.61	409.48	No	Yes
PSF113_3994	-	Hypothetical protein	7.75	737.07	No	Yes
PSF113_4041	-	Hypothetical protein	11.1	1508.18	Yes	Yes
PSF113_4078a	-	Hypothetical protein	5	355.54	No	No
PSF113_4247	-	Putative membrane protein, clustering with ActP	5.96	515.64	No	No

PSF113_4468	-	Hemolysin activator protein precursor	6.56	706.08	Yes	No
PSF113_4474	-	Hypothetical protein	9.39	1210.6	Yes	Yes
PSF113_4849	-	Hypothetical protein	6.17	527.26	No	Yes
PSF113_5053	-	Hypothetical protein	10.01	1677.23	Yes	Yes
PSF113_5145	-	Hypothetical protein	8.8	1127.39	No	Yes
PSF113_5316	-	Hypothetical protein	12.74	2187.83	Yes	Yes
PSF113_5726b	-	Hypothetical protein	7.56	1213.02	No	Yes
**VIRULENCE**						
PSF113_0435	-	N-acetylglucosamine-binding protein A	8.25	1400.62	Yes	Yes
PSF113_1855	-	RHS repeat-associated core domain-containing protein	11.78	2113.91	Yes	Yes
PSF113_2409	*vgrG*	VgrG	12.24	1568.85	Yes	Yes
PSF113_2470	-	Insecticidal toxin	8.1	795.51	No	Yes
PSF113_3048	-	Polyketide synthase	5.51	395.33	No	Yes
PSF113_5062	-	Lantibiotic mersacidin modifying enzyme	5.81	686.95	No	No
PSF113_5817	*icmF2*	IcmF2	8.19	1106.94	No	Yes

Supplementary Table 4. 218 genes putatively regulated by FleQ in *Pseudomonas fluorescens* F113 *sadC⁻ wspRC⁻*. Genes likely regulated by FleQ in *Pseudomonas fluorescens* F113 *sadC⁻ wspRC⁻* background found in ChIP-seq analysis with a fold enrichment value equal or higher than five were chosen. Selected genes were classified in functions according to Gene Ontology. The table includes the locus, gene and protein name, two statistical values: fold enrichment and –log10 (q-value) that indicate the probability of certainty of a binding site to be found; and the comparison with *Pseudomonas fluorescens* F113 ChIP-seq and *Pseudomonas fluorescens* F113 *bifA⁻* ChIP-seq assays. q-value = False discovery rate (FDR).

LOCUS	GENE	PRODUCT	FOLD ENRICHMENT	-log10(qvalue)	GENE FOUND IN F113 ChIP-seq	GENE FOUND IN F113 bifAChIP-seq
**c-di-GMP**						
PSF113_3487	-	Diguanylate phosphodiesterase	15.8	2247.57	Yes	Yes
PSF113_4023	-	Diguanylate cyclase phosphodiesterase with PAS/PAC sensor	10.02	1187.58	Yes	Yes
PSF113_5478	-	Response regulator sensory box GGDEF domain EAL domain-containing protein	6.16	582	Yes	Yes
PSF113_5738	-	GGDEF domain/EAL domain protein	10.47	1573	Yes	Yes
**CELL WALL**						
PSF113_0208	*lapA*	LapA	7.59	979.93	Yes	Yes
PSF113_0453a	-	Putative glutamate racemase	9.89	1324.6	Yes	No
PSF113_1644	*wzz*	Wzz	8.61	853.39	Yes	Yes
PSF113_4777	*lpxC*	LpxC	5.01	386.34	No	Yes
PSF113_5774	-	Putative lipoprotein	7.49	943.42	No	No
**IRON**						
PSF113_0198	*foxI*	FoxI	7.7	1240.19	No	Yes
PSF113_0933	*fagA*	FagA	15.02	2173.51	Yes	No
PSF113_1274	-	TonB-dependent hemin, ferrichrome receptor	6.63	882.43	Yes	Yes
PSF113_1322	-	Iron-regulated protein A precursor	7.2	830.22	Yes	Yes
PSF113_1749	*pvdS*	PvdS	11.49	1370.8	Yes	Yes
PSF113_1837	*pvdD*	PvdD	12.92	1738.36	Yes	Yes
PSF113_1841	-	Outer membrane (iron.B12.siderophore.hemin) receptor	12.12	1493.03	No	No
PSF113_1856	-	Outer membrane pyoverdine efflux protein	5.95	479.7	Yes	Yes
PSF113_1867	-	RNA polymerase sigma-70 factor, ECF subfamily	17.43	2603.05	Yes	Yes
PSF113_1869	-	FecA	5.81	454.07	No	Yes
PSF113_2258	-	Outer membrane ferripyoverdine receptor	6.81	566.26	Yes	Yes
PSF113_2454	-	RNA polymerase sigma-70 factor, ECF subfamily	7.61	689.79	Yes	Yes
PSF113_2456	-	Ferrichrome-iron receptor	9.37	984.07	Yes	Yes
PSF113_2589	-	Ferrichrome-iron receptor	5.02	318.93	Yes	Yes
PSF113_2837	-	RNA polymerase sigma-70 factor, ECF subfamily	8.17	779.49	Yes	Yes
PSF113_3151	-	Ferrichrome-iron receptor	17.09	2604.76	Yes	Yes
PSF113_3153	-	RNA polymerase sigma-70 factor, ECF subfamily	5.33	358.94	Yes	Yes
PSF113_3220	-	Heme uptake regulator	5.86	430	Yes	Yes
PSF113_3435	-	Cytochrome P450	15.81	2251.15	No	No
PSF113_3734	-	Ferrichrome-iron receptor	5.68	404.87	Yes	Yes
PSF113_3792	-	Periplasmic protein p19 involved in high-affinity Fe2+ transport	6.31	673.38	No	Yes
PSF113_4045	-	Iron-regulated membrane protein	10.52	1353.51	Yes	Yes
PSF113_4536	-	Putative thiol oxidoreductase with 2 cytochrome c heme-binding sites	13.58	1782.4	Yes	Yes
PSF113_4568	-	Bacterioferritin-associated ferredoxin	9.95	1944.11	Yes	Yes
PSF113_4845	-	RNA polymerase sigma-70 factor, ECF subfamily	15.09	2401.77	Yes	Yes
PSF113_4896	-	Ferrichrome-iron receptor	14.76	2087.86	Yes	Yes
PSF113_4980	-	Iron abc transporter	7.41	694.31	No	No
PSF113_4983	-	Periplasmic hemin-binding protein	7.41	694.31	No	Yes
PSF113_5410	*fiuI*	FiuI	8.59	911.25	No	Yes
PSF113_5411	*fiuR*	FiuR	13.25	2286.19	Yes	No
PSF113_5657	*fbpA*	FbpA	11.47	2037.49	Yes	Yes
PSF113_5672	-	TonB-like protein	5.47	650.85	No	Yes
PSF113_5691	-	Cytochrome c family protein	10.14	1608.97	Yes	Yes
**MOTILITY/CHEMOTAXIS**						
PSF113_0199	-	Methyl-accepting chemotaxis protein	7.7	1240.19	No	Yes
PSF113_0569	-	Methyl-accepting chemotaxis protein	8.55	1295.17	Yes	Yes
PSF113_0751	*flhD*	FlhD	8.72	1550.59	Yes	No
PSF113_1434	-	Methyl-accepting chemotaxis protein	9.1	1400.18	No	Yes
PSF113_1554	*fliC*	FliC	5.4	367.69	Yes	No

PSF113_1562	*fliE*	FliE	12.04	2105.01	Yes	Yes
PSF113_1572	*fliL*	FliL	11.45	1363.38	Yes	Yes
PSF113_1579	*flhB*	FlhB	8.7	914.44	No	Yes
PSF113_2159	-	Methyl-accepting chemotaxis protein	10.77	1235.81	Yes	Yes
PSF113_2925	-	Methyl-accepting chemotaxis protein	8.09	767.65	No	Yes
PSF113_4456	*cheV*	CheV	11.57	1400.59	Yes	Yes
PSF113_4457	*flgA*	FlgA	11.57	1400.59	Yes	Yes
PSF113_5017	-	Methyl-accepting chemotaxis protein	10.55	1781.3	Yes	Yes
**OTHERS**						
PSF113_0079b	-	Putative restriction endonuclease	5.08	419.1	Yes	Yes
PSF113_0079c	-	Phage-related replication protein-like protein	5.52	479.4	Yes	Yes
PSF113_0139	*trpB*	TrpB	7.19	640.1	No	Yes
PSF113_0159	-	NADH:ubiquinone oxidoreductase subunit 2 (chain N)	5.43	537.92	Yes	Yes
PSF113_0265	-	Acyl-CoA dehydrogenase, probable dibenzothiophene desulfurization enzyme	5.42	893.72	No	No
PSF113_0351	-	Yibq gene product, possible divergent polysaccharide deacetylase	7.31	889.86	Yes	Yes
PSF113_0422	-	Penicillin-binding protein 1A	6.52	722.38	No	No
PSF113_0572	-	3'-to-5' exoribonuclease RNase R	7.37	735.31	Yes	Yes
PSF113_0624	*urtD*	UrtD	5.11	495.58	No	Yes
PSF113_0711	-	Exodeoxyribonuclease V gamma chain	7.85	1309.57	Yes	Yes
PSF113_0869	*nusA*	NusA	5.22	416.3	Yes	Yes
PSF113_0870	*infB*	InfB	7	851.24	No	Yes
PSF113_0889	-	Flavodoxin nitric oxide synthase	11.65	1972.69	Yes	Yes
PSF113_1047	-	Multicopper oxidase	7.81	1080.82	Yes	Yes
PSF113_1069	*yaeQ*	YaeQ	6.35	498.35	No	Yes
PSF113_1201	-	Ferredoxin--NADP(+) reductase	6.09	520.96	Yes	Yes
PSF113_1216	-	Methylated-DNA--protein-cysteine methyltransferase-related protein	9.64	1346.12	No	Yes
PSF113_1217	-	Peptide chain release factor RF-3	9.64	1346.12	No	Yes
PSF113_1490	-	Filamentous hemagglutinin, intein-containing, putative	14.81	2429.67	No	No
PSF113_1491	-	Transposase-like protein	16.71	2557.33	Yes	Yes
PSF113_1511	-	Putative hemagglutinin/hemolysin-related protein	5.06	324.56	Yes	Yes
PSF113_1514	*recQ*	RecQ	13.89	1846.11	No	No
PSF113_1657	-	Acyltransferase 3	5.72	410.99	No	Yes
PSF113_1658	-	O-antigen acetylase	5.72	410.99	No	Yes
PSF113_1815	-	Integral membrane protein	6.51	526.3	Yes	Yes
PSF113_1835	-	Trans-aconitate 2-methyltransferase	7.22	628.86	No	No
PSF113_1836	-	Putative thioesterase involved in non-ribosomal peptide biosynthesis	7.22	628.86	No	Yes
PSF113_1919	-	Oxidoreductase, short chain dehydrogenase/reductase family	6.13	562.82	No	Yes
PSF113_1923	-	FAD dependent oxidoreductase	6.13	562.82	No	Yes
PSF113_2058	*arsH*	ArsH	7.31	642.66	No	Yes
PSF113_2103	-	Phenylpropionate dioxygenase-related ring-hydroxylating dioxygenase, large terminal subunit	14.55	2411.85	Yes	Yes
PSF113_2126	-	Dihydrodipicolinate synthase	10.66	1216.23	Yes	Yes
PSF113_2158	*nuoA*	NuoA	10.77	1235.81	Yes	Yes
PSF113_2611	*pdtF*	PdtF	14.15	2390.2	Yes	Yes
PSF113_2636	-	Multidrug RND efflux membrane fusion protein	15.87	2263.11	Yes	Yes
PSF113_2646	*mutT*	MutT	10.52	1190.6	No	Yes
PSF113_2698	*ligD*	LigD	7.49	671.35	No	Yes
PSF113_2926	-	Aminotransferase class I and II	8.09	767.65	No	Yes
PSF113_2972	-	Glycosaminoglycan degradation	14.36	1943.84	Yes	Yes
PSF113_3123	-	Serine protease, subtilase family	14	1870.13	No	No
PSF113_3156	-	Dihydrofolate reductase	5.33	358.94	Yes	Yes
PSF113_3162	-	Dienelactone hydrolase-related enzyme	6.26	485.56	No	Yes
PSF113_3307	-	Acetate kinase	9.9	1135.24	No	Yes
PSF113_3308	-	Rossmann fold nucleotide-binding domain-containing protein	8.69	891.8	No	Yes
PSF113_3422	*nosR2*	NosR2	11.9	1649.51	No	Yes
PSF113_3504	-	Transposase IS3/IS911 family protein	6.8	564.6	Yes	Yes
PSF113_3571	*phnB*	PhnB	8.59	849.66	Yes	Yes
PSF113_3785	-	Ribonucleotide reductase of class III (anaerobic), large subunit	5.62	702.04	No	Yes
PSF113_3795	-	Putative collagenase	6.17	473.66	No	Yes
PSF113_3889	-	Zinc carboxypeptidase domain protein	13.08	2030.98	Yes	Yes
PSF113_3918	*tig*	Tig	8.89	965.72	Yes	Yes
PSF113_3922	*folD*	FolD	8.89	965.72	Yes	Yes

PSF113_4083	-	Sterol desaturase	13.37	2302.93	Yes	Yes
PSF113_4204	-	Protein binding	8.39	1206.13	Yes	Yes
PSF113_4230	*htpG*	HtpG	7.53	838.62	No	No
PSF113_4280	-	DNA mismatch repair protein	5.63	474.75	No	Yes
PSF113_4311	-	Pyocin holin	5.7	479.17	No	No
PSF113_4393	-	Imidazoleglycerol-phosphate synthase	6.34	497.55	No	Yes
PSF113_4394	-	Imidazoleglycerol-phosphate synthase	6.6	770.22	Yes	Yes
PSF113_4478	*rsmA*	RsmA	9.97	1102.66	Yes	Yes
PSF113_4523	-	Mercuric reductase	9.12	1169.87	Yes	Yes
PSF113_4532	-	Translation initiation factor 2 (IF-2, GTPase)	5.57	420.04	No	No
PSF113_4567	-	Alkyl hydroperoxide reductase subunit C-like protein	9.95	1944.11	Yes	No
PSF113_4717	-	Ribonuclease III	5.34	363.88	No	No
PSF113_4848	-	Ribosomal subunit interface	6.14	590.79	No	Yes
PSF113_4882	-	4-aminobutyrate aminotransferase	5.41	369.16	No	No
PSF113_4932	*prs*	Prs	9.88	1381.99	Yes	Yes
PSF113_4952	-	Carbonic anhydrase	5	362.73	No	No
PSF113_4953	-	Phosphotransferase system, HPr-related protein	13.56	2303.94	No	No
PSF113_4961	-	Paraquat-inducible protein A	5.47	391.14	Yes	No
PSF113_4979	-	Regulator of competence-specific genes	5.47	391.14	No	Yes
PSF113_4984	-	Rieske 2Fe-2S family protein	7.93	980.21	No	Yes
PSF113_5096	-	4-hydroxyphenylpyruvate dioxygenase	11.44	1484.48	No	Yes
PSF113_5146	*leuS*	LeuS	5.49	432.05	No	Yes
PSF113_5200	-	Threonine synthase	5.49	432.05	No	Yes
PSF113_5203	*metZ*	MetZ	8.57	1080.79	No	No
PSF113_5322	*tyrZ*	TyrZ	14.24	2364.97	No	Yes
PSF113_5389	-	Stress induced hydrophobic peptide	6.92	718.17	No	Yes
PSF113_5415	*creD*	CreD	5.01	378.24	No	No
PSF113_5479	-	Alginate lyase precursor	6.16	582	Yes	Yes
PSF113_5482	*fda*	Fda	11.65	1926.23	Yes	Yes
PSF113_5626	*spuA*	SpuA	8.56	1140.86	Yes	Yes
PSF113_5627	*spuI*	SpuI	8.56	1140.86	Yes	Yes
PSF113_5684	*ppx*	Ppx	5.16	423.84	No	No
PSF113_5685	*ppk*	Ppk	5.16	423.84	No	No
PSF113_5739	*rep*	Rep	10.47	1573	Yes	Yes
PSF113_5844	-	Homocysteine S-methyltransferase	9.98	1377.94	No	Yes
PSF113_5892	*wbpZ*	WbpZ	5.63	511.06	No	No
**REGULATION/SIGNAL TRANSDUCTION**						
PSF113_0138	*trpI*	TrpI	7.19	640.1	No	No
PSF113_0563	*hfq*	Hfq	5.15	407.52	No	Yes
PSF113_0990a	-	Transcriptional regulator, TetR family	5.71	523.87	No	Yes
PSF113_0993	-	Sefir domain protein	6.73	931.89	No	No
PSF113_1200	-	LysR family transcriptional regulator	6.09	520.96	Yes	Yes
PSF113_1439	-	Transcriptional regulator, LysR family	5.3	384.68	No	Yes
PSF113_1719	-	DNA-binding response regulator, LuxR family	6.72	553.8	No	Yes
PSF113_1897	-	Transcriptional regulator, TetR family	5.91	510.83	Yes	Yes
PSF113_1927	-	Transcriptional regulator, AraC family	8.3	1020.5	No	Yes
PSF113_2256	-	Transciptional regulator	5.15	348.84	No	Yes
PSF113_2728	-	Transcriptional regulator, TetR family	5.12	331.64	No	Yes
PSF113_3264	-	Transcriptional regulator, IclR family	15.11	2100.34	No	Yes
PSF113_3439	*pnpR*	PnpR	15.81	2251.15	No	Yes
PSF113_4024	-	Transcriptional regulator, Cro/CI family	10.02	1187.58	Yes	Yes
PSF113_4373	-	DNA recombination-dependent growth factor C	9.26	1657	Yes	Yes
PSF113_4792	*mraZ*	MraZ	5.05	385.25	No	Yes
PSF113_5280	*rplR*	RplR	12.43	1860.01	No	Yes
PSF113_5301	*rpoC*	RpoC	7.98	911.39	No	Yes
**TRANSPORT**						
PSF113_0209	-	Type I secretion outer membrane family	7.59	979.93	Yes	Yes
PSF113_0904	-	Dipeptide-binding ABC transporter, periplasmic substrate-binding component	5.73	482.51	No	No
PSF113_1510	-	Family type I secretion outer membrane protein	5.06	324.56	Yes	Yes
PSF113_1945	-	Permeases of the major facilitator superfamily	16.94	2496.45	Yes	Yes
PSF113_1972	*attE*	AttE	15.79	2246.37	Yes	Yes

PSF113_2040	-	Sugar ABC transporter (ATP-binding protein)	7.97	747.75	Yes	Yes
PSF113_2676	*nhaB*	NhaB	16.97	2576.75	No	Yes
PSF113_3830	-	Low-affinity inorganic phosphate transporter	7.45	664.36	No	No
PSF113_3993	-	ABC transporter, ATP-binding/permease protein	14.38	1947.31	No	Yes
PSF113_4178	*rcpA*	RcpA/CpaC	6.16	1353.36	Yes	Yes
PSF113_4199	-	Transporter, LysE family	8.39	1206.13	Yes	Yes
PSF113_4377	-	C4-dicarboxylate transporter/malic acid transport protein	5.37	429.3	No	Yes
PSF113_4522	*aroP*	AroP	9.12	1169.87	Yes	Yes
PSF113_4581	*gltI*	GltI	6.72	579.33	No	Yes
PSF113_5308	*secE*	SecE	10.81	1980.51	No	Yes
PSF113_5673	*glpT*	GlpT	5.47	650.85	No	Yes
PSF113_5845	-	Amino acid ABC transporter, periplasmic amino acid-binding protein	9.98	1377.94	No	Yes
**UNKNOWN**						
PSF113_0145	-	Hypothetical protein	14.46	2357.91	No	Yes
PSF113_0455b	-	Putative membrane protein	7.37	1029.37	No	No
PSF113_0799	-	Hypothetical protein	5.03	523.85	Yes	Yes
PSF113_0800	-	Transmembrane protein	6.51	806.01	Yes	Yes
PSF113_0905	-	Hypothetical protein	5.73	482.51	No	No
PSF113_0928	-	Putative exported protein	9.51	1008.46	No	Yes
PSF113_0990	-	4-oxalocrotonate tautomerase family protein	5.71	523.87	No	Yes
PSF113_1427	-	Hypothetical protein	5.07	393.43	Yes	Yes
PSF113_1438	-	Hypothetical protein	5.3	384.68	No	Yes
PSF113_1648	-	Hypothetical protein	5.3	355.3	Yes	No
PSF113_1651	-	Glycosyl hydrolase BNR repeat-containing protein	6.7	567.63	No	Yes
PSF113_1656	*wbpL*	WbpL	5.7	517.65	No	Yes
PSF113_1937	-	hypothetical protein	11.11	1299.19	No	Yes
PSF113_1944	-	Transmembrane protein	16.94	2496.45	Yes	Yes
PSF113_2272	-	Hypothetical protein	7.5	672.23	Yes	Yes
PSF113_2273	-	Reticulocyte binding protein	7.5	672.23	Yes	Yes
PSF113_2470	-	Insecticidal toxin	17.19	2550.34	No	Yes
PSF113_2522	-	Hypothetical protein	5.65	413.6	No	Yes
PSF113_2642	-	Putative membrane protein	8.77	879.69	No	No
PSF113_2645	-	Putative exported protein	8.77	879.69	No	Yes
PSF113_2938	-	Putative membrane protein	5.08	325.97	Yes	Yes
PSF113_2956	-	Hypothetical protein	6.2	549.47	No	Yes
PSF113_3171	-	Hypothetical protein	7.37	652.18	No	Yes
PSF113_3311	-	Putative membrane protein	9.53	1011.4	No	Yes
PSF113_3793	-	Hypothetical protein	6.31	673.38	No	Yes
PSF113_3994	-	Hypothetical protein	14.38	1947.31	No	Yes
PSF113_4041	-	Hypothetical protein	9.94	1344.31	Yes	Yes
PSF113_4474	-	Hypothetical protein	9.97	1102.66	Yes	Yes
PSF113_4849	-	Hypothetical protein	6.14	590.79	No	Yes
PSF113_5053	-	Hypothetical protein	5.39	410.48	Yes	Yes
PSF113_5059	-	Putative exported protein	6.51	528.5	No	No
PSF113_5145	-	Hypothetical protein	11.44	1484.48	No	Yes
PSF113_5316	-	Hypothetical protein	14.24	2364.97	Yes	Yes
PSF113_5726b	-	Hypothetical protein	7.51	1225.47	No	Yes
**VIRULENCE**						
PSF113_0324	-	Hypothetical protein	8.87	1475.1	Yes	Yes
PSF113_0435	-	N-acetylglucosamine-binding protein A	14.64	2393.98	Yes	Yes
PSF113_1855	-	RHS repeat-associated core domain-containing protein	5.95	479.7	Yes	Yes
PSF113_2409	*vgrG*	VgrG	6.57	531.59	Yes	Yes
PSF113_3048	-	Polyketide synthase	5.72	463.11	No	Yes
PSF113_5817	*icmF2*	IcmF2	9.04	1237.58	No	Yes

Supplementary Table 5. 162 genes putatively regulated by FleQ in *Pseudomonas putida* KT2440 *bifA*⁻. Genes putatively regulated by FleQ in *Pseudomonas putida* KT2440 *bifA*⁻ background obtained from ChIP-seq analysis with a fold enrichment value equal or higher than five were selected. Genes were fuctionaly classified according to Gene Ontology. The table includes the locus, gene and protein name, two statistical values: fold enrichment and –log10 (q-value) that indicate the probability of certainty of a binding site to be found; and the comparison with *Pseudomonas putida* KT2440 ChIP-seq. q-value = False discovery rate (FDR).

LOCUS	GENE	PRODUCT	FOLD ENRICHMENT	-log10(qvalue)	GENE FOUND IN KT2440 ChIP-seq
**c-di-GMP**					
PP_0131	-	Membrane-anchored phosphodiesterase	6.46257	15.94667	Yes
PP_0563	*gcbA*	Two-component system response regulator	6.10947	13.44655	Yes
PP_5263	-	GGDEF domain-containing protein	13.90545	55.68275	Yes
**CELL WALL**					
PP_0168	*lapA*	LapA	27.08751	155.38925	Yes
PP_0504	*oprG*	OprG	5.3544	10.25368	Yes
PP_1288	*algD*	GDP-mannose 6-dehydrogenase	10.09264	32.38295	Yes
PP_1795	-	Polysaccharide synthesis, Peb	5.7763	11.91402	Yes
PP_1970	-	Lipoprotein	19.66527	79.53717	Yes
PP_2003	-	Putative lipoprotein	5.53805	10.26796	No
PP_2191	-	Lipoprotein	13.83852	47.57621	Yes
PP_2629	*pseudogene*	Pseudogene (first in Bcs operon)	6.31231	13.04807	Yes
PP_3016	-	Lipopolysaccharide core biosynthesis protein	6.55509	13.97967	No
PP_3126	-	Polysaccharide export protein, Pea	7.76899	18.8671	Yes
PP_3733	-	ABC transporter	6.08055	12.56435	Yes
PP_4057	-	Outer membrane autotransporter	5.2839	10.10284	Yes
PP_4097	*pgsA*	CDP-diacylglycerol--glycerol-3-phosphate 3-phosphatidyltransferase	5.3155	10.17087	No
**IRON**					
PP_0160	*foxA*	Ferrioxamin receptor	25.09126	128.69612	Yes
PP_0162	-	ECF family RNA polymerase sigma-70 factor	21.67727	109.79546	Yes
PP_0180	-	Cytochrome c family protein	8.88578	28.85159	Yes
PP_0267		Ferric siderophore receptor	8.70604	27.29845	Yes
PP_0350	*fiuA*	Ferrichrome-iron receptor	6.76569	15.75471	Yes
PP_0352	-	ECF family RNA polymerase sigma-70 factor	8.33384	22.66193	Yes
PP_0482	*bfrA*	Bacterioferritin	6.03604	13.28321	No
PP_0535	-	TonB-dependent siderophore receptor	7.38079	20.43962	Yes
PP_0667	-	ECF family RNA polymerase sigma-70 factor	10.26759	34.61795	Yes
PP_0700	-	Transmembrane sensor	5.8233	12.80551	No
PP_0701	*fsrI*	Fosmidomycin resistance protein / MFS efflux transporter	5.8233	12.80551	No
PP_0704	-	ECF family RNA polymerase sigma-70 factor	9.1226	26.63008	Yes
PP_0861	-	Outer membrane ferric siderophore receptor	15.87369	66.57471	Yes
PP_1006	-	Heme receptor	11.39012	40.51083	Yes
PP_1083	-	BFD (2Fe-2S)-binding domain-containing protein	32.0137	171.15695	Yes
PP_2192	-	ECF family RNA polymerase sigma-70 factor/transmembrane sensor prot	13.83852	47.57621	Yes
PP_2590	-	Outer membrane ferric siderophore receptor	30.59042	146.8309	Yes
PP_3086	-	ECF subfamily RNA polymerase sigma-70 factor	8.9829	24.09186	Yes
PP_3612	-	TonB-dependent siderophore receptor	6.31231	13.04807	Yes
PP_4611	-	ECF family RNA polymerase sigma-70 factor	40.76645	237.02948	Yes
PP_4755	-	TonB-dependent siderophore receptor	27.80478	142.29903	Yes
PP_4881	-	Iron ABC transporter substrate-binding protein	5.33914	10.94871	Yes
**MOTILITY/CHEMOTAXIS**					
PP_2128	*cheV-like*	CheV-like chemotaxis protein	6.06953	12.13372	No
PP_2454	*rbsB*	RbsB	5.82675	11.23725	No

PP_3557	-	methyl-accepting chemotaxis transducer	5.80747	11.19966	No
PP_4335	*motB*	Flagellar motor protein MotD	29.06982	141.29414	Yes
PP_4339	*cheZ*	CheZ	6.54017	14.81798	No
PP_4344	*flhA*	Flagellar biosynthesis protein FlhA	36.15101	203.02502	Yes
PP_4352	*flhB*	Flagellar biosynthesis protein FlhB	9.91893	31.37439	Yes
PP_4361	*fliK*	Flagellar hook-length control protein	7.34165	19.38266	Yes
PP_4370	*fliE*	Flagellar hook-basal body protein FliE	44.60622	269.69067	Yes
PP_4373	*fleQ*	FleQ	25.35785	184.51305	No
PP_4376	*fliD*	FliD	20.0656	97.69353	No
PP_4378	*fliC*	FliC	8.01797	24.5142	No
PP_4386	*flgF*	Flagellar basal-body rod protein FlgF	20.85595	97.94153	Yes
PP_4391	*flgB*	Flagellar basal-body rod protein FlgB	38.06229	218.97942	Yes
PP_4393	*cheV-3*	Chemotaxis protein CheV	23.58743	115.525	Yes
PP_4394	*flgA*	Flagellar basal body P-ring biosynthesis protein FlgA	23.58743	115.525	Yes
PP_4395	*flgM*	Anti-sigma-28 factor FlgM	5.29462	10.84827	Yes
PP_4888	-	Methyl-accepting chemotaxis sensory transducer	11.16531	38.67562	Yes
**OTHERS**					
PP_0117	*znuB*	Hypothetical protein	35.31078	198.71301	Yes
PP_0420	*trpG*	Anthranilate synthase component II	8.57459	24.81134	Yes
PP_0437	*birA*	Biotin-protein ligase	25.44057	129.57225	Yes
PP_0440	*tuf*	Elongation factor Tu	8.21052	24.99614	Yes
PP_0545	*aldB-I*	Aldehyde dehydrogenase	9.25832	28.07472	Yes
PP_0597	*mmsA-1*	methylmalonate-semialdehyde dehydrogenase	24.98844	126.51683	No
PP_0626	*ndh*	Ndh	10.86124	36.04551	No
PP_0680	-	ATP-dependent protease La Type II	10.90558	38.67453	No
PP_0705	*alkA*	DNA-3-methyladenine glycosylase II	9.1226	26.63008	Yes
PP_0722	*prs*	Ribose-phosphate pyrophosphokinase	5.7019	12.52801	No
PP_0788	-	Hypothetical protein	7.23276	18.17844	Yes
PP_1064	-	Alpha/beta fold family hydrolase	5.08667	9.32998	No
PP_1084	-	Anti-oxidant AhpCTSA family protein	32.0137	171.15695	Yes
PP_1100	*dcd*	Deoxycytidine triphosphate deaminase	6.10918	14.27143	No
PP_1157	-	Acetolactate synthase	24.32837	117.32132	Yes
PP_1207	-	Histidine triad (HIT)	17.57011	79.55122	Yes
PP_1296	*estB*	Carboxylesterase	6.13578	13.91941	No
PP_1702	*rdgC*	DNA recombination-dependent growth factor C	5.96085	11.91014	No
PP_1703		Assimilatory nitrate reductase large subunit	5.96085	11.91014	No
PP_1785	*rmlB*	dTDP-glucose 4,6-dehydratase	7.89683	21.66267	Yes
PP_1786	-	Glycosil transferase family protein	7.89683	21.66267	Yes
PP_1878	-	Hypothetical protein	9.03601	24.76163	Yes
PP_1957	-	Pdr/VanB family oxidoreductase	5.23966	9.65045	No
PP_1969	*moaA2*	MoaA2	19.66527	79.53717	No
PP_2132	-	Universal stress protein	5.34118	9.50095	No
PP_2239	*rhtA*	Cysteine transporter	27.03288	137.43167	Yes
PP_2453	*ansA*	Asparaginase/glutaminase	5.82675	11.23725	No
PP_2477	*iorA-I*	Isoquinoline 1-oxidoreductase subunit alpha	6.06953	12.13372	No
PP_3015	-	Medium-chain acyl-CoA ligase domain-containing protein	5.58396	10.35992	No
PP_3184	*mcoA*	Mn(II) copper oxidase A	6.79787	14.92687	Yes
PP_3239	*tnpT-II*	Cointegrate resolution protein T	6.13807	13.0961	No
PP_3324	-	GTP cyclohydrolase I type 2	8.49734	21.9645	Yes
PP_3547	-	Short chain dehydrogenase/reductase oxidoreductase	12.13905	38.96114	Yes
PP_3569	*quiA*	Quinate dehydrogenase	5.72242	11.02475	Yes
PP_3700	*parI*	Chromosome partitioning ATPase	32.31284	186.7485	Yes
PP_3947	-	Nicotinate dehydrogenase subunit A	5.50733	10.57623	No
PP_4070	*panC*	Pantothenate synthetase	7.09778	18.80794	Yes

PP_4264	*hemN1*	Coproporphyrinogen III oxidase, oxygen-independent	6.05157	12.50306	No
PP_4350	-	Aminotransferase	9.91893	31.37439	Yes
PP_4459	-	Transposase	15.8061	66.40699	Yes
PP_4469	*gmk-1*	Phosphonate metabolism protein/1,5-bisphosphokinase PhnN	8.92497	26.72785	Yes
PP_4610	-	PepSY-associated TM helix domain-containing protein	40.76645	237.02948	No
PP_4674	*recC*	Exodeoxyribonuclease V subunit gamma	31.19625	173.33627	Yes
PP_4714	*rimP*	Ribosome maturation factor	7.65469	22.08843	No
PP_4880	*vacB*	Ribonuclease R	5.33914	10.94871	Yes
PP_5045	*thiI*	Thiamine biosynthesis protein ThiI	9.21768	27.97917	Yes
PP_5046	*glnA*	Glutamine synthetase, type I	9.21768	27.97917	Yes
PP_5264	*rep*	ATP-dependent helicase Rep	13.90545	55.68275	Yes
**REGULATION/SIGNAL TRANSDUCTION**					
PP_0298	*gbdR*	GbdR / AraC family transcriptional regulator	11.85458	42.95822	No
PP_0546	-	Fis famility transcriptional regulator	9.25832	28.07472	Yes
PP_0625	*clpB*	ATP-dependent Clp protease, ATP-binding subunit ClpB	10.86124	36.04551	Yes
PP_1099	*cspA2*	CspA	6.10918	14.27143	No
PP_1637	-	LysR family transcriptional regulator	11.20013	36.83834	Yes
PP_1651	-	Two-component system response regulator	10.83681	35.36063	Yes
PP_1978	-	TetR family transcriptional regulator	9.71124	27.37209	Yes
PP_3185	*pet18*	TenA famility transcriptional regulator	6.79787	14.92687	Yes
PP_3238	-	Transcriptional regulator PyrR	6.13807	13.0961	No
PP_3503	-	Fis family transcriptional regulator	5.0984	8.66449	Yes
PP_4345	-	GntR family transcriptional regulator	36.15101	203.02502	Yes
PP_4363	*rsbU*	RsbU / response regulator receiver protein	6.16402	13.56734	No
PP_4470	*amrZ*	Arc domain-containing protein DNA binding domain-containing protein	8.92497	26.72785	Yes
**TRANSPORT**					
PP_0167	*paxB*	LapA type I secretion system ATPase	27.08751	155.38925	Yes
PP_0268	-	Porin	8.70604	27.29845	Yes
PP_0503	-	Major facilitator transporter	5.01677	9.18137	No
PP_1002	*arcD*	Arginine/ornithine antiporter	6.13476	14.33088	Yes
PP_1156	-	Formate/nitrate transporter	24.32837	117.32132	Yes
PP_1185	*oprH*	Outer membrane protein H1	6.34315	13.53519	Yes
PP_1206	*oprD*	Porin	17.57011	79.55122	Yes
PP_1297	*yhdW*	Amino acid ABC transporter-binding protein YhdW	6.13578	13.91941	No
PP_1728	-	Hypothetical protein	8.9829	24.09186	Yes
PP_1797	-	HlyD family secretion protein	5.7763	11.91402	Yes
PP_2240	-	ABC transporter ATP-binding protein	27.03288	137.43167	No
PP_3789	-	Efflux transporter	6.58413	14.47626	Yes
PP_4458	-	Opine ABC transporter substrate-binding protein	15.8061	66.40699	Yes
PP_4471	*mgtE*	Magnesium transporter	11.48754	40.74831	Yes
PP_4495	*aroP-II*	Aromatic amino acid transport protein	5.91771	12.61796	No
PP_4519	*tolC*	TolC-type I secretion outer membrane protein	18.32678	88.92484	Yes
PP_4643	-	Xanthine/uracil permease family protein	5.1123	10.08143	No
**UNKNOWN**					
PP_0018	-	Hypothetical protein	7.54554	21.32738	No
PP_0020	-	Hypothetical protein	7.94836	22.80224	No
PP_0153	-	Hypothetical protein	5.66356	12.05401	No
PP_0534	-	Winged helix family two component transcriptional regulator	7.38079	20.43962	Yes
PP_0599	*pqiB*	Hypothetical protein	24.98844	126.51683	Yes
PP_0685	-	Hypothetical protein	8.5478	24.21119	Yes
PP_0862	-	Hydroxylase	15.87369	66.57471	Yes
PP_1149	-	Hypothetical protein	8.51979	23.61195	Yes
PP_1865	-	ISPpu8, transposase	19.84879	90.2562	Yes
PP_1958	-	Hypothetical protein	5.23966	9.65045	No

PP_2114	-	ISPpu8, transposase	7.04065	15.88995	Yes
PP_2115	-	Hypothetical protein	9.22568	25.17389	No
PP_2133	-	Hypothetical protein	5.34118	9.50095	No
PP_2219	-	Hypothetical protein	25.73479	115.85802	Yes
PP_2473	-	Hypothetical protein	5.80269	11.97132	Yes
PP_2509	-	Hypothetical protein	7.28343	16.86853	Yes
PP_2688	-	Hypothetical protein	5.0984	8.66449	No
PP_3011	-	Hypothetical protein	6.06953	12.13372	Yes
PP_3504	-	Hypothetical protein	5.0984	8.66449	Yes
PP_3685	-	Hypothetical protein	5.33648	10.21553	Yes
PP_3696	-	Hypothetical protein	5.60513	11.16066	Yes
PP_3704	-	Hypothetical protein	5.27995	10.09454	No
PP_3784	-	Hypothetical protein	5.1071	9.02685	No
PP_4096	-	Hypothetical protein	5.3155	10.17087	No
PP_4317	-	Hypothetical protein	42.39947	260.50143	Yes
PP_4328	-	Hypothetical protein	5.2045	9.57641	No
PP_4739	-	Hypothetical protein	6.10932	13.85958	No
**VIRULENCE**					
PP_2563	-	Antibiotic biosynthesis protein	5.89127	12.55978	No

Supplementary Table 6. Oligonucleotides used in RT-qPCR experiment. List and sequence of primers used for RT-qPCR assays in *Pseudomonas fluorescens* F113 (PSF113_) and *Pseudomonas putida* KT2440 (PP_).

Locus	Gene	Forward primer	Reverse primer
PSF113_1554	*fliC*	5'-GTAAACACTAACGTCACATCG-3	5'-ATACGGGTAGCGATCTGTAAG-3
PSF113_4470	*amrZ*	5'-GAAGTGGCTCGCAATCATCAC-3	5'-CATGCTCAACTCTTCACCCAA-
PSF113_2409	*vgrG*	5'-GACAGCAACACCACGCTATCA-3	5'-CGTTGAG-GATGTGCGAACC-3
PSF113_0208	*lapA*	5'-GGTTTTCGTCGTATCCCCAGA-	5'-CAGCCAGATCCAAGTGACG-3
PSF113_1970	-	5'-CCTCTACGGCTATCGCAAGGA-	5'-AAAATTCTTCCACTCGGTGCAG-
PSF113_1750	*pvdL*	5'-AGGCAGGAAACCCCCATGAC-3	5'-CGCGATAACTCAACACCACAC-3
PSF113_1749	*pvdS*	5'-CGCCAGAAACCTCCATATCA-3	5'-GCGGTACATCTCGAAGGCATAG-3
PSF113_1274	-	5'-CCACCTGAAAAAGCCCTGGA-3	5'-GCGCTGATGGTCACTTGTTG-
PSF113_3220	-	5'-TCCGCCAACGATCTGTCTCTA-	5'-ACGTATCCTGGCAAGGTCTG-
PP_0168	*lapA*	5'-GTCGATAGCTCGGGCAGTATG-	5'-CGACCAGGAAGATGTTTACGG-3
PP_2635	*bcsA*	5'-GTTTCCCGACTTTGATGCTGA-	5'-GAATAGGTTTCTGCCGCCAAC-
PP_4378	*fliC*	5'-ACTCTGTCGCTGTTCGCTGAC	5'-CCGTGATGTTCAGGTTGGAAA-3
PP_4470	*amrZ*	5'-CTGAACTGTCCCTGCATGAGC-	5'-GCCATTTCCACATCGTGAGC-
PP_2590	-	5'-GGGCTTATGTGGAAACCAAGG-3	5'-CGATAGTCCGTCTTGCTGGTG
PP_4606	-	5'-CGGCGGTGTATGACTTTGGTA-	5'-GGCAGGTCGAAGGTATTGGTC-
PP_4611	-	5'-TTACCACGCACACCACAACTG-3	5'-AGGCGCATGTAGGTGTCCTG-
-	*16S rRNA*	5'-TCAGTCACACTGGAACTGAG-3	5'-CAGGCCGTCAACTTAATGCG-
